# Daytime lighting conditions alter home-cage behavior across both sexes in diurnal grass rats

**DOI:** 10.3389/fnins.2026.1882564

**Published:** 2026-09-17

**Authors:** Jeremiah Hartner, Noah Muscat, Mujtaba Khan, Daniil Berezhnoi, Katrina Linning-Duffy, Diksha Zutshi, Nicolette Ognjanovski, Lily Yan, Brendon O. Watson

**Affiliations:** 1 Watson Laboratory, Department of Psychiatry, University of Michigan, Ann Arbor, MI, United States; 2 Yan Laboratory, Department of Psychology, Michigan State University, East Lansing, MI, United States

**Keywords:** actigraphy, *Arvicanthis niloticus*, circadian rhythms, diurnal, Major Depressive Disorder, Nile grass rat, Seasonal Affective Disorder

## Abstract

Circadian rhythms are crucial to biological functions, and cognitive functions such as attention, choice, and preference-related behaviors are modulated by circadian rhythms and disrupted in mood disorders such as Seasonal Affective Disorder (SAD) and Major Depressive Disorder (MDD). These neuropsychiatric diseases can be induced or worsened by alterations to daily light patterns and can also be treated with circadian-timed bright-light therapy, suggesting modulatory effects of light brightness on mood and behavior. While most laboratory rodents are nocturnal, the Nile grass rat (*Arvicanthis niloticus*) is diurnal, offering a unique model to study light modulation effects relevant to humans. In this work, we track daily activity in male and female grass rats under varied lighting for several weeks, revealing sex-specific circadian patterns and responses. These findings establish a foundation for mechanistic studies of light effects on mood-related brain circuits in diurnal animals.

## 1 Introduction

Circadian rhythms are crucial to basic daily functions, and disruptions or alterations to these rhythms are implicated in changes to brain health seen in neuropsychiatric disease states. Numerous functions, including cognition, attention, emotionality and preference-related behaviors are modulated by circadian rhythms (Fujisawa et al., 2008; Manning et al., 2019; Ferenczi et al., 2016; Kim et al., 2016). Further, Seasonal Affective Disorder (SAD) and Major Depressive Disorder (MDD) show a strong circadian rhythm in symptomology and can also be worsened or induced by disturbance in circadian cycles or light alterations (Vogel et al., 2012; Costa, 2010; Swanson et al., 2018; Burgess et al., 2004; Monteleone et al., 2011; Wirz-Justice, 2008). In addition, circadian-timed light therapy can be an effective treatment for both SAD (Rosenthal et al., 1984; Terman and Terman, 2005) and non-seasonal depression, suggesting modulatory effects of light on mood regardless of season (Monteleone et al., 2011; Terman and Terman, 2005). This also suggests that brain circuits are modulated over the 24-h cycle, which we have recently shown electrophysiologically (Ognjanovski et al., 2025), and are also affected by light, especially mood-related regions such as the medial prefrontal cortex (mPFC). Elucidating how mood-related circuits are impacted by circadian rhythms and daily light exposure is crucial to understanding human circadian brain biology, but methods to study those detailed dynamics are too invasive to use in humans, and so rodents provide a valuable experimental model.

Efforts to understand the biology of daily light rhythms in a manner most relevant to humans are hindered by the fact that commonly used laboratory rodents are nocturnal while humans are diurnal. While the central molecular circadian pacemaker is conserved in both function and phase between nocturnal and diurnal animals, in diurnal animals, light promotes wakefulness/arousal and can be therapeutic in humans (Rosenthal et al., 1984; Terman and Terman, 2005); while in nocturnal animals, light induces sleep (Borbély, 1976). Though nocturnal animal behavior generally appears to be anti-phase to diurnal animal behavior in response to light exposure, identical stimuli can elicit different behavioral responses between diurnal and nocturnal animals (Shuboni et al., 2012). Further, the organization of circadian oscillator systems in nocturnal versus diurnal species differs in a more complex manner than a simple flip in the daily behaviors despite in-phase SCN pacemakers, as many but not all extra-SCN oscillators are phase reversed (Challet, 2007; Yan et al., 2020; Smale et al., 2003). Thus, studies of nocturnal models cannot be directly translated or simply reversed to understand functions of diurnal brains and their response to altered lighting conditions. In fact, numerous therapeutic agents proven effective in nocturnal rodent models failed in human clinical trials (Esposito et al., 2020; Cederroth et al., 2019), which reveal a translational gap in our understanding of the neurophysiologic effects of light on diurnal brains.

The Nile grass rat (NGR; *Arvicanthis niloticus*) is diurnal in the wild (Blanchong and Smale, 2000) and is now a well-established laboratory model exhibiting diurnal patterns of sleep, locomotor activity, mating behavior, body temperature and secretion of luteinizing hormones in the laboratory (McElhinny et al., 1997; Novak et al., 1999; Mahoney and Smale, 2005; McElhinny et al., 1999). Further, NGRs show light-dependent behavioral and brain responses and have shown characteristics consistent with SAD in reduced lighting conditions (Costello et al., 2023). Like patients, NGRs also show brightness-related behavioral alterations (Kim et al., 2023) and in fact standard laboratory lighting conditions, which provide much less light than natural daylight may induce depressed behavior. For example, NGRs limited to a dimly lit environment in the daytime exhibit reduced sweet solution preference behavior (Leach et al., 2013; Lonstein et al., 2019), while NGRs exposed to bright light in the daytime show improved spatial memory (Yan et al., 2019; Soler et al., 2018), indicating that NGR physiology and behavior are similarly affected by variations in lighting conditions. However, there have not been attempts to quantify movement over longer time scales involving continuous recordings over many months in varying lighting conditions.

The NGR may potentially offer several specific advantages over nocturnal rodent models for studying SAD and light-related mood disorders. Most critically, NGRs and humans share increased activity patterns in response to light, meaning that both species are active during the light phase and rest during the dark phase. This diurnal pattern has important mechanistic implications because light promotes wakefulness and arousal in diurnal species, whereas the same stimulus induces sleep in nocturnal animals (Borbély, 1976). Thus, the NGR response to light exposure and the consequences of altered environmental stimuli are aligned with those observed in humans in a manner that nocturnal models cannot replicate. NGRs exposed to reduced daytime lighting exhibit reduced sucrose preference (Leach et al., 2013; Lonstein et al., 2019), a well-validated test for anhedonia and one of the principal symptoms of both SAD and MDD. Further, bright light improves cognitive performance in NGRs (Yan et al., 2019; Soler et al., 2018), which parallels the therapeutic effects of light therapy in humans (Rosenthal et al., 1984; Terman and Terman, 2005).

Despite these advantages, important translational limitations should be acknowledged. The NGR is not a naturally occurring model of SAD but rather a species whose diurnal behavior makes it susceptible to lighting-induced activity changes analogous to those seen in humans. Unlike humans, NGRs do not have a documented seasonal history of affective episodes, and the neuroendocrine and hormonal factors governing their light sensitivity may differ from those in humans in ways that are not yet fully characterized. Furthermore, the artificial vivarium environment includes fixed 12:12 light–dark cycles, single housing, limited spectral range of LED lighting, and stark, non-graded, lighting transitions which introduce constraints that limit direct extrapolation to the complex natural and social environments in which human circadian disruption occurs. These limitations are important for interpreting the present findings and may motivate future studies to implement more naturalistic environmental and social conditions.

Given prior evidence that NGR behavior is sensitive to light intensity (Kim et al., 2023; Leach et al., 2013; Lonstein et al., 2019), we approached this study with a primary hypothesis that exposure to bright light (>1,000 lux) would increase daytime-specific activity and enhance diurnality compared to standard vivarium lighting (300 lux). We further predicted that, as a diurnal species with a strong endogenous circadian clock, NGRs would show anticipatory activity ahead of daily light transitions. Although grass rats are fundamentally diurnal, prior work has shown they can also exhibit crepuscular activity, with peaks around both the dark-to-light and light-to-dark transitions (Costello et al., 2023; Caputo et al., 2022; Caputo et al., 2025). We therefore predicted anticipatory ramping during dawn and dusk transitions. We additionally predicted that in the absence of light entrainment (constant darkness condition), NGRs would maintain a near-24-h free-running rhythm reflecting a strong endogenous circadian clock.

Interestingly, sex-specific 24-h behavior patterns in various lighting conditions have not been examined. This is especially important given the contribution of circadian rhythms and light to mental health (Fujisawa et al., 2008; Manning et al., 2019; Ferenczi et al., 2016; Kim et al., 2016), and the known human sex differences in susceptibility to anxiety disorders, MDD, and SAD (Altemus et al., 2014; Kessler et al., 2012; Sramek et al., 2016; Kornstein et al., 2000; Jalnapurkar et al., 2018). Because of the underrepresentation of female animals in prior NGR studies, we chose to include both sexes to ensure an accurate representation of the NGR model. Thus, we set out to establish an experimental system to objectively assess male and female NGR movement in various lighting conditions within the home-cage over several months. We did not explicitly design a study to compare males and females, but rather, we performed sex-based comparisons as a secondary, exploratory analysis after noticing variable response patterns in the individual animal data.

In this project, we quantify the daily actigraphy of both male and female diurnal rodents over extended periods of time in (1) standard laboratory 12:12 h light/dark (LD) cycle (300 lux in daytime) (2) more naturalistic/brighter LD (1,000 lux daytime) and (3) constant darkness (DD; 0 lux) “free-run” conditions to assess daily and circadian rhythmicity. We quantify results by tracking changes in videographic pixel values from frame to frame. We aim to reveal the rhythms of daily activity across lighting conditions in diurnal mammals using a novel tracking technique to improve our understanding of behavior in rodents that share diurnality with humans. This can in turn establish a novel experimental paradigm to study the biology of SAD and/or light therapy.

## 2 Materials and methods

### 2.1 Subjects

Male and female Nile grass rats (*Arvicanthis niloticus*) were acquired from the well-established breeding colony at Michigan State University managed by Yan et al. (2019). Animals were transported to the University of Michigan at 8–12 weeks of age and allowed to acclimate to the new facility for a minimum of 3 weeks prior to testing. Animals are initially group-housed in standard Plexiglas cages (17″ × 9″ × 8″) and provided nesting materials and hay for enrichment. Animals are transferred to single housing a minimum of 1 week prior to experiment start. Animals were single-housed throughout the study. Single housing was required both for unambiguous per-animal quantification of pixel-based activity, which cannot resolve individual animals when they are co-housed, and to accommodate planned *in vivo* electrophysiological recordings in this cohort, which require individual housing.

Animals were housed with a 12:12 h light–dark (LD) cycle under standard vivarium lighting (300 lux as measured from the center of the cage on the top-rack of vivarium shelving <4 feet from the overhead ceiling lights) with water and food (Prolab RMH 2000 5P06, Brentwood, MO, United States) ad libitum. Our vivarium lighting measures 100–300 lux depending on location of the animal cage within the housing rooms relative to the overhead lighting. The animals in this study were all housed on upper racks with a light intensity of 300 lux, which is consistent with an assessment of other research institution typical vivarium lighting conditions (Dauchy and Blask, 2023; Patel et al., 2021). Standard vivarium lighting was provided by overhead fluorescent fixtures emitting broadband white light with color temperature of approximately 4,000 K and a spectral range of approximately 400–700 nm, measured at 300 lux at animal eye level using a calibrated light meter (Extech Industrial Electronics, LT300, Knoxville, TN, United States) within the cage for each of the 4 cage positions on the top rack of vivarium shelving. For our bright light conditions, we added LED overhead rack-mounted LED tube lighting (Dong Guan City Shengwei Lighting Co., Ltd., Dongguan, CN; 20 W, 2400 lumens, 5,000 K daylight white, T8 form factor) to raise the illuminance to 1,000–1,300 lux (as measured at NGR eye level) with the goal of having the animal experience light intensities more like what a wild NGR might experience in outdoor lighting conditions considering direct sunlight is generally obscured by NGR tendency to burrow in tall grasses or soil. Ambient temperature was maintained at a constant setpoint by the facility’s centralized HVAC system throughout the study and across all lighting conditions. Supplemental bright-light illumination was provided by LED fixtures, which produce minimal radiant heat, such that lighting condition did not appreciably affect cage temperature.

Male and female NGRs were randomly assigned a cage position on the top rack of the vivarium shelving, and standard cage tops were modified using wire mesh (304 stainless steel, plain weave, 1.3 mm aperture, 0.15 mm wire diameter) to allow overhead camera capture with clear visibility of the animal. Four animals in four individual cages were recorded continuously at 12 frames per second 24 h per day using StreamPix software (Norpix, Montreal, CA) paired with monochrome cameras (Ace U acA1300-200um, Basler, Exton, PA, United States) with 6 mm, ½” lenses (Edmund Industrial Optics, Barrington, NJ, United States) and bandpass near infrared filters: 845 to 860 nm (Midwest Optical Bi850-43, Graftek Imaging, Austin, TX, United States). for the duration of the experiment. Camera was triggered to record for 59:59 min at a time and then re-started every 60 min.

Each cohort of animals was recorded over a period lasting 2–3 months, beginning with 5 weeks (an initial one-week habituation period followed by 4 weeks of baseline data collection) in standard vivarium 300 lux LD conditions, followed by 4 weeks of exposure to 1,000–1,300 lux LED lighting during the lights phase of the LD cycle. The experiment finished with 4 weeks in constant dark (DD) lighting at approximately 0 lux, achieved by enclosing the cages under blackout curtains for 24 h per day to shield the animals from exposure to the vivarium room lighting operating on a 12:12 h light–dark cycle at ~300 lux during the lights-on phase (Figure 1). Illuminance inside the enclosed cages measured 0 lux, indicating light levels at or below the meter’s detection threshold. We cannot entirely rule out minor, intermittent light exposure, such as brief curtain displacement or light during routine cage changes; this exposure was not quantified. However, it is not considered to have influenced the observed activity patterns, as all animals expressed free-running rhythms under this condition, indicating that any residual light was insufficient to serve as a zeitgeber for this diurnal species. We therefore use the term “constant darkness (DD),” standard in the circadian literature for a free-running condition. Detailed spectral power distribution and irradiance were not directly measured for either the standard vivarium fluorescent lighting or the supplemental LED lighting used in this study; only illuminance (lux) was quantified. The experiment consisted of 3 cohorts of 4 animals each for a total of 12 animals (6M/6F). The first cohort (2M/2F) were not exposed to the DD condition. The 2nd and 3rd cohort were exposed to all lighting conditions (Table 1). All recorded experimental days from all animals were fully analyzed and included in the results. All procedures were conducted following the guidelines set by the Institutional Animal Care and Use Committee (IACUC) of the University of Michigan.

**Figure 1:**
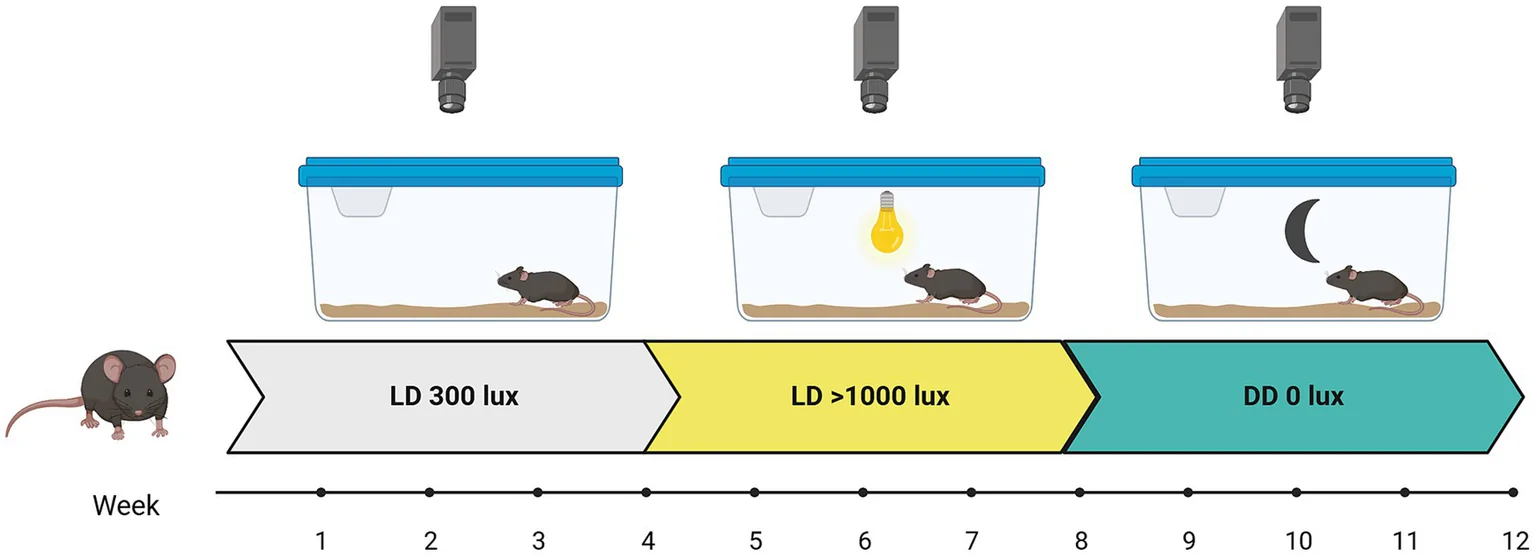
Experimental timeline. Schematic of recording protocol over the course of 3 months. Some animals completed all 3 phases of the protocol (*n* = 8), a subset were recorded in only LD 300 lux and LD 1000 lux conditions (*n* = 4). Created in BioRender. Hartner, J. (2026) https://BioRender.com/ybmrx7e.

**Table 1:** Experimental design by animal.

Animal ID	Sex	Cohort	Rack location	Condition 1: LD 300 lux	Condition 2: LD ≥ 1,000 lux	Condition 3: DD 0 lux
AO1	M	1	Left	Y	Y	N
AO2	M	1	Mid left	Y	Y	N
AO3	M	1	Mid right	Y	Y	N
AO4	F	1	Right	Y	Y	N
AO5	F	2	Left	Y	Y	Y
AO6	F	2	Mid left	Y	Y	Y
AO7	M	2	Mid right	Y	Y	Y
AO8	F	2	Right	Y	Y	Y
AO9	M	3	Left	Y	Y	Y
AO10	F	3	Mid left	Y	Y	Y
AO11	F	3	Mid right	Y	Y	Y
AO12	M	3	Right	Y	Y	Y

For each animal, Y denotes exposure to the indicated lighting condition and N indicates no exposure.

### 2.2 Actigraphy

Actigraphy analysis was performed by developing Python-based code^1^ to extract number of pixels showing changes in brightness over time, frame-to-frame, and was used as a proxy for animal movement. To achieve this, a simple script was created with several steps including grayscale conversion, dual-threshold filtering (global thresholding and percentage change thresholding), dilation, and region size filtering. This script tracks only pixels that exceed the thresholds for each step (details below), requiring that spatially contiguous groups of a minimum number of pixels show change in brightness, which minimizes noise and selects changes likely due to animal movement. The output provides a Selected Pixel Difference (SPD) value, which is the count of all pixels per frame that meet the selection criteria. By incorporating timestamps with each video frame, we then use SPD as a proxy for animal movement over time.

To compute the pixel-wise changes frame-to-frame, we first convert each pixel value from color to grayscale, which gives a single brightness value that can be more simply tracked frame-to-frame. We then perform dual-threshold filtering, where the change in brightness value must exceed both a global threshold and percentage change threshold compared to the previous frame. All pixels that meet both criteria are converted to binary (white or black), and only white pixels are kept. We then dilate areas of white pixels to fill in small gaps between neighboring white pixels. This creates multiple blobs—one for each area of the pixel intensity change—from which we can find the one corresponding to the moving animal. The size of each of the contiguous areas of white pixels is calculated and a user-defined threshold is used to eliminate smaller white regions which are unlikely to be representative of pixel changes due to animal movement. The final output is the SPD value representing the total number of white pixels in each frame.

Each of the thresholding parameters is user-defined through a graphical user interface (GUI), which allows for iterative testing to refine pixel change tracking. This is best achieved by using a second script to provide video output highlighting all selected pixels in each frame based on the user-defined parameters (Figures 2A,B). Thus, the user can alter the thresholds for pixel changes and immediately assess the quality of animal tracking. Once pixel change threshold parameters are optimized, the script can be run on all videos within that same environment without further calibration, and further refinements can be made for different video environments or varying light conditions. Standard default thresholds are as follows: minimum size threshold = 100, global threshold = 30, percentage threshold = 35, dilation kernel = 5.

**Figure 2:**
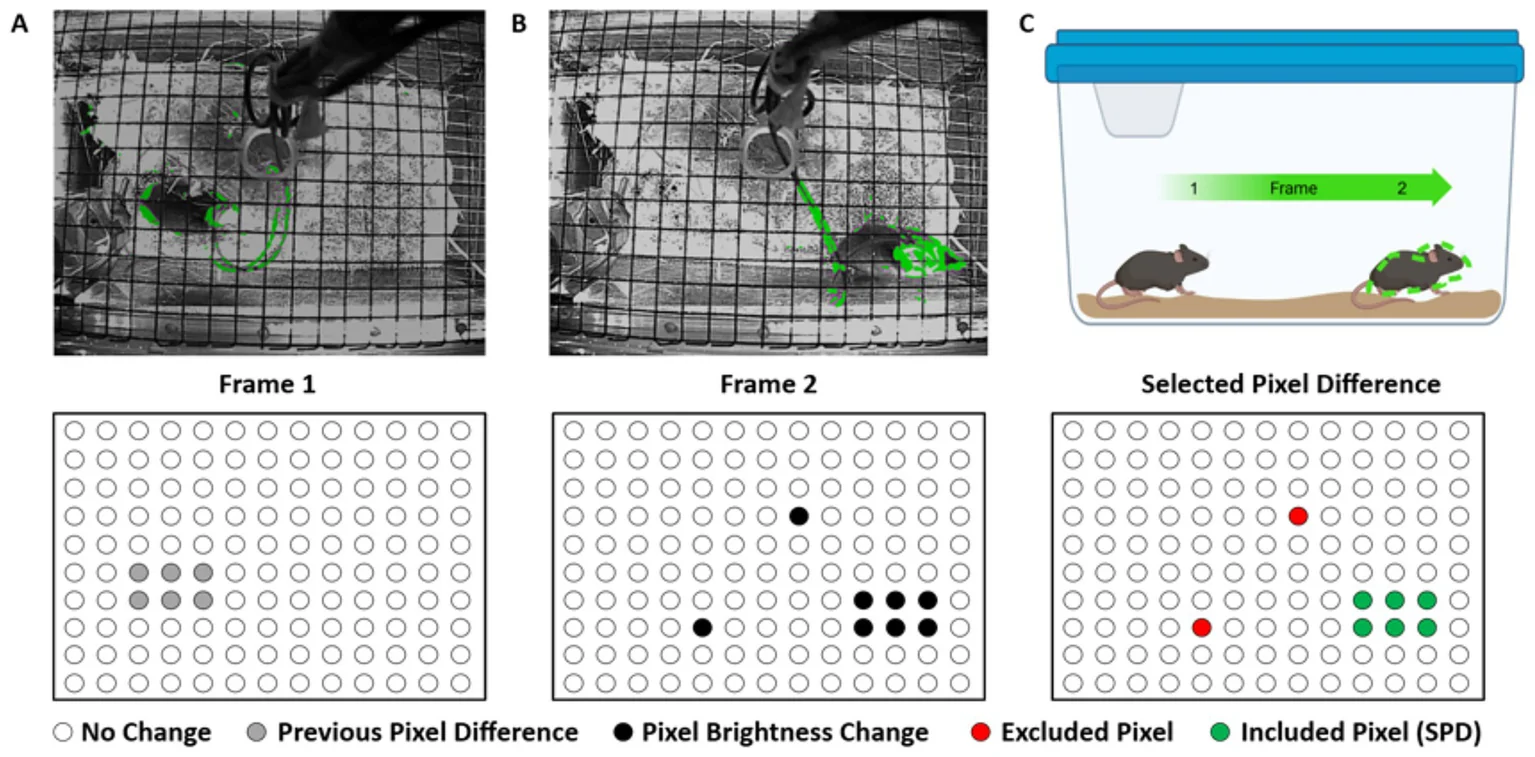
Selected pixel difference as a measure of animal movement. **(A)** Top: Demonstrative example. Frame 1 shows the animal’s current position as tracked using frame-to-frame actigraphy. Frame-to-frame selected pixel changes are represented in green. Bottom: Schematic illustrating pixel changes within the captured camera frame. **(B)** Top: Example frame 2 showing animal movement to the opposite side of the cage. Actigraphy SPD tracking represented in green. Bottom: Schematic illustrating pixel changes within the captured camera frame. **(C)** Top: Representative output of SPD as the animal traverses the cage. Bottom: Schematic demonstrating exclusion of pixel brightness changes to refine SPD tracking of animal-related movement frame-to-frame. Created in BioRender. Hartner, J. (2026) https://BioRender.com/ybmrx7e.

After calibrating parameters for multiple animals in different lighting conditions, a single set of parameters (minimum size threshold = 160, global threshold = 30, percentage threshold = 35, dilation kernel = 3) optimally tracked our animals’ movement over time irrespective of animal, cage conditions, lighting, or cage placement within the room (Figure 2). Tracking of other animal types in varying environments may need different parameters, though this was not tested. These parameters were then applied to all videos from all animals in the experiment, allowing us to efficiently pull selected pixel difference from each frame of continuous months-long video recordings. We then used these pixel changes as a proxy for animal movement to compare hourly rhythms of activity across animals and lighting conditions over time. The output of the script is a single .csv file showing the SPD value for every time-stamped video frame for every animal across all conditions.

To assess the validity of the SPD metric as a proxy for locomotor activity, we performed several validation steps. First, our approach follows the same established frame-differencing principle used in validated open-source rodent home-cage monitoring systems, in which locomotor activity is quantified from frame-to-frame changes in pixel intensity within the video (Singh et al., 2019); this principle also underlies widely used open-source behavioral-tracking frameworks such as Bonsai (Lopes et al., 2015). Second, visual inspection of video recordings was conducted alongside SPD traces for multiple animals across all three lighting conditions, confirming that periods of high SPD corresponded to visible whole-body movement and that periods of low SPD corresponded to behavioral quiescence or sleep. Third, to independently validate that our implementation of the algorithm accurately tracks the animal’s position, we manually scored a subset of frames from the video data and determined the XY coordinates of the center of mass for the animal. There was a strong correlation between our manually labeled XY coordinates and the XY coordinate of the pixel changes from the output of our script (centroid of the SPD object, Pearson’s r for x-coordinate = 0.96, for y-coordinate = 0.80). Finally, we also calculated total SPD (used in the article) and total XY distance (summed Euclidean distance) for 1-h bins in one animal over multiple days, and the resulting data showed high correlation between those two measurements (Pearson’s r = 0.95; Supplementary Figure S1), indicating that SPD can accurately track both the position and movement of the animal.

Data are output as .csv files and to enable locking to clock time and therefore subsequently lights-on Zeitgeber time, each frame is timestamped in POSIX timestamps based on video file start time plus frames elapsed multiplied by seconds-per-frame.

### 2.3 Statistical analyses

Data analysis was performed using MATLAB (R2023a, MathWorks Inc.) using the selected pixel difference (SPD) as the measure of activity. All timestamps were converted to Zeitgeber time (ZT) with lights-on defined as ZT0 for each animal. Changes in SPD were compared to a baseline/control condition to track changes in animal movement across minutes or hours for each of the lighting conditions. For each lighting condition, activity was quantified from the final 14 days of the 300 lux and 1,000 lux conditions and the final 7 days of the constant darkness (DD) condition (the shorter window for DD reflecting the shifting of animals’ subjective day/night in the absence of light entrainment), and compared to the individual animal’s baseline movement in 300 lux. The final 14 days were chosen to allow the animal multiple weeks to acclimatize to the new lighting condition and control for any stress response likely to be seen immediately following an environmental alteration. The resulting datasets were examined by comparing binned averages of SPD in bins of 5 min or 60 min across days and weeks to determine significant differences in movement across time in different lighting conditions.

Statistical comparisons were assigned to one of three tiers according to their pre-specified role. We pre-planned three primary hypotheses and reported them without multiple-comparisons correction: that bright light increases daytime activity and enhances diurnality, that the NGR free-running period approximates 24 h due to a strong endogenous clock, and that NGRs show anticipatory movement during both daily light transitions. Exploratory and secondary comparisons were corrected using the Benjamini-Hochberg false-discovery-rate procedure (q = 0.05) within their respective families, as detailed under Multiple comparisons below. Standardized effect sizes and 95% confidence intervals for all primary comparisons are provided in Supplementary Table 1. Effect sizes and confidence intervals.

Standardized effect sizes with 95% confidence intervals were computed for all primary comparisons directly from the per-animal data. For paired (within-animal) *t*-tests, we report Cohen’s d_z, defined as the mean of the within-animal differences divided by the standard deviation of those differences. For independent (between-group) *t*-tests, we report Hedges’ g, calculated as Cohen’s d (mean difference divided by the pooled standard deviation) multiplied by the small-sample correction factor J = 1–3/(4(n₁ + n₂) − 9); the uncorrected Cohen’s d is also reported. For the one-sample comparison of the free-running period against 24 h, we report Cohen’s d (mean deviation divided by the standard deviation). Ninety-five percent confidence intervals for all effect sizes were obtained using the normal approximation. For the linear mixed-effects models, we report both the unstandardized fixed-effect coefficient (*β*, in pixel-difference units) with its 95% confidence interval and a standardized effect defined as *β* divided by the total model standard deviation (the square root of the sum of the between-animal variance and the residual variance).

#### 2.3.1 Multiple comparisons

Correction for multiple comparisons was applied according to the pre-specified role of each analysis. Pre-planned primary hypotheses, namely that bright light increases daytime activity and enhances diurnality, that the free-running period approximates 24 h, and that animals anticipate both daily light transitions, were tested as planned comparisons and are reported without correction. Exploratory analyses were corrected using the Benjamini-Hochberg false-discovery-rate procedure (q = 0.05) within their respective families: the hour-by-hour activity-profile comparisons were corrected across the 24 hourly bins, and the between-condition transition comparisons were corrected within their family. Sex-based comparisons, which were secondary and conducted *post hoc* after inspection of individual-animal variability, were likewise FDR-corrected within each analysis family. All statistical analyses were performed in MATLAB.

#### 2.3.2 Diurnality index

A diurnality index was calculated to simplify activity differences seen during the light phase compared to the dark phase. DI is calculated by using the percentage change in raw (not normalized) SPD during the light phase compared to dark phase for each of the lighting conditions:


$$\mathrm{DI}=\frac{\left(\text{light phase}\;\mathrm{SPD}-\text{dark phase}\;\mathrm{SPD}\right)}{\text{dark phase}\;\mathrm{SPD}}x100$$

#### 2.3.3 Linear mixed effects model

The choice between *t*-tests and linear mixed-effects models was determined by the structure of each comparison: analyses that retained multiple daily observations per animal were analyzed with linear mixed-effects models to account for within-animal correlation, whereas comparisons reduced to a single summary value per animal per condition (e.g., mean mid-phase activity, diurnality index, free-running period, and anticipatory activity) were analyzed with paired or independent *t*-tests, for which no additional within-animal variance structure remains to be modeled. To compare activity levels between lighting conditions while accounting for the repeated measures structure of the data, we used linear mixed effects models (LME) in MATLAB (fitlme) as an alternative to ANOVA due to the repeated measures of animals over varying numbers of days. Each model included lighting condition as a fixed effect and individual animal as a random intercept, allowing each animal to have its own baseline activity level. This approach uses all daily observations (approximately 14–30 days per animal per condition) while accounting for the non-independence of days recorded within the same animal. Model fit was assessed using Akaike Information Criterion (AIC). Fixed effects were tested using *t*-statistics with Satterthwaite degrees of freedom approximation as implemented by MATLAB’s fitlme function. For between-sex comparisons, sex was included as the fixed effect with individual animal as a random intercept.

## 3 Results

### 3.1 Daily movement patterns in standard vivarium lighting

We first confirmed NGRs show diurnal patterns of movement in our laboratory setting over time. To test this, animals were individually moved to cages with overhead cameras and were allowed to acclimatize to that for at least 1 week prior to testing. A 12-week paradigm was then used to test the effects of daytime light intensity as shown in Figure 1 exposing animals to LD (300 lux), LD (1,000 lux) and DD (0 lux) for 4 weeks each. Quantification of daily movement was done using the SPD metric outlined in the methods. A total of 12 single-housed NGRs (6 male, 6 female) were recorded in 3 separate cohorts of 4 animals each (Table 1).

To assess circadian rhythmicity, a Lomb-Scargle Periodogram was performed (plomb’ function in MATLAB) on 5 min bins of SPD spanning the final 14 days of the baseline LD (300 lux) condition evaluated over periods from 0.5 to 36 h. The last 14 days were chosen as the analysis window to account for any stress while acclimating to the new environment along with a noticeable settling of animal activity during the first 2 weeks (Supplementary Figure S3A). Each animal’s binned activity was z-scored prior to pooling so that animals with higher overall activity did not dominate the mean spectrum. The resulting periodogram showed NGR activity was consistently cyclical with a large peak near both 12 and 24 h, indicating a strong daily cycle of movement patterns (Figure 3A). Pooled actogram and 24-h activity profile analyses of SPD across the last 14 days of standard 300 lux laboratory lighting (*n* = 12 NGRs) showed clear increases in activity during the light phase (lights ON, ZT0-11) relative to the dark phase (lights OFF, ZT12-23) with a pronounced peak at the light-to-dark transition (ZT11-12) and a secondary rise near ZT6 (Figures 3B,C). Comparing each hour against the mean of the remaining hours (per-hour paired *t*-tests, Benjamini-Hochberg FDR-corrected across the 24 hourly bins, q = 0.05), 12 of the 24 hourly bins reached significance (FDR-adjusted *p* < 0.0231). Activity was significantly increased during the light phase and around the light-to-dark transition (ZT5, ZT10-12) and significantly reduced across the dark phase (ZT15-21 and ZT23; Figures 3B,C).

**Figure 3:**
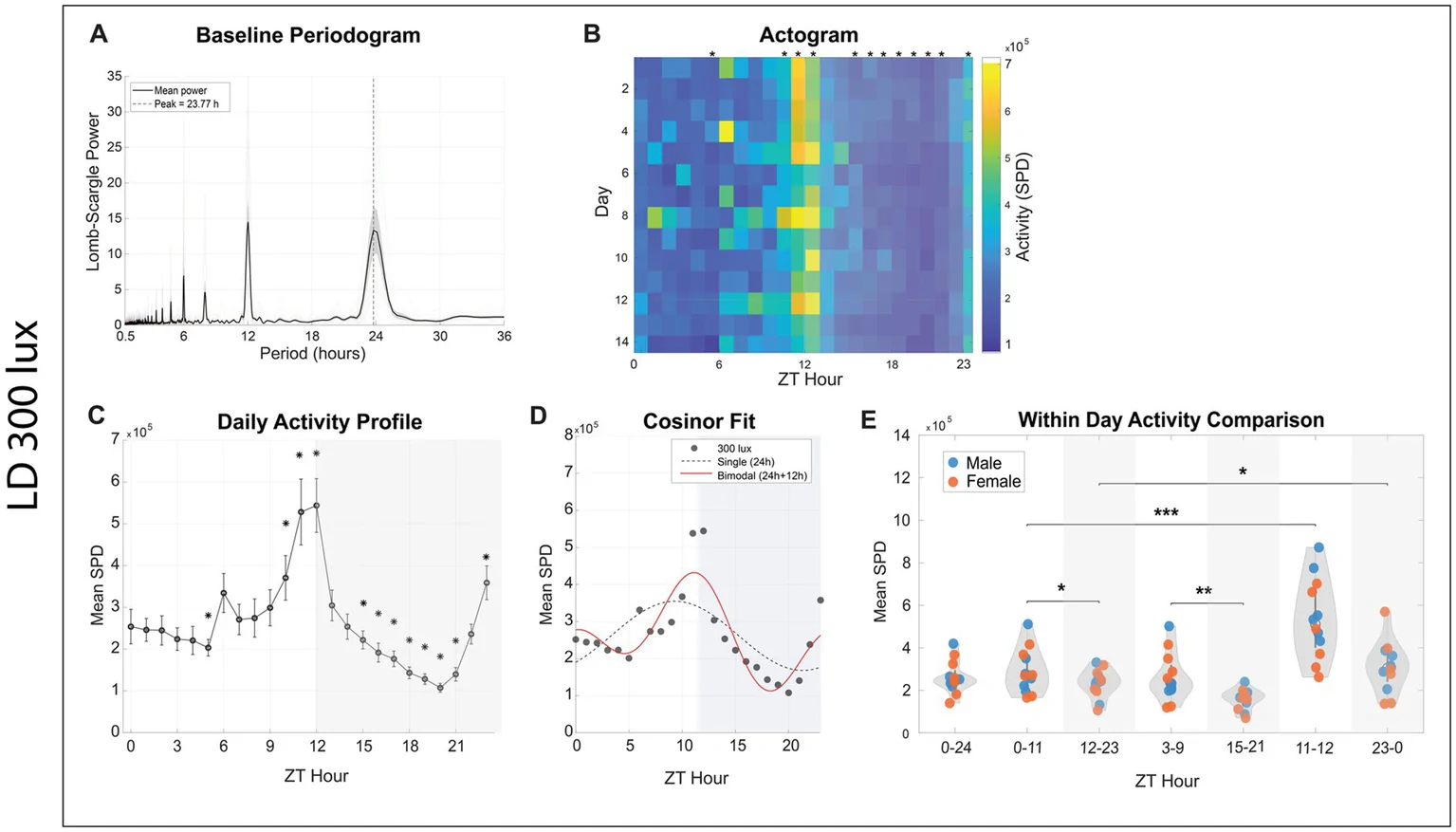
Characterization of animal movement using selected pixel difference (SPD). NGRs display diurnal patterns of activity. **(A)** Periodogram of SPD data testing for rhythmicity and patterns of movement across the final 14 days of baseline 300 lux conditions. Peaks indicate a repeating pattern of activity at a given lag. Animal activity shows a clear peak around 24 h indicating patterned animal movement on a circadian scale. **(B)** Actogram analysis detailing average pixel change by hour (*n* = 12) across the final 14 days of recording at 300 lux daytime light. 300 lux is considered standard laboratory conditions. Degree of pixel change representing animal activity demonstrated by heat-map (yellow indicates more pixel change). Left portion of plot (lights-on hours) generally shows more activity than right (lights-off hours). Asterisks above hourly columns indicate hours that differ significantly from the average of the other 23 h (per-hour *t*-tests, Benjamini-Hochberg false-discovery-rate corrected across the 24 hourly bins, *q* = 0.05). **(C)** Mean selected pixel difference for all animals by hour across the final 14 days of the 300 lux baseline condition (averaged first within animal for each hour across days, then across animals). Activity shows peaks in mean activity at ZT12 and 23 as well as more activity during lights ON vs. OFF (left vs. right side), matching the actogram analysis. Asterisks indicate hours that differ from the distribution of all 23 other hours (per-hour *t*-tests, Benjamini-Hochberg false-discovery-rate corrected, *q* = 0.05): Activity peaks around ZT12 and reaches minimum around ZT15–21. **(D)** Cosinor analysis of the pooled baseline activity profile under 300 lux. The mean hourly activity (selected pixel difference, SPD; filled circles) across the final 14 days of the 300 lux condition is overlaid with a single-component (24-h; black dashed) and a bimodal (24-h + 12-h harmonic; red solid) Cosinor fit of the cross-animal averaged hourly means. Grey shading indicates the dark phase (lights off, ZT12–23). Activity was significantly rhythmic with a dominant 24-h component peaking during the light phase (acrophase ZT9.3), and the bimodal model significantly improved the fit over the single-component model (nested *F*-test, *p* = 0.0004), indicating a significant crepuscular (12-h) component near the light transitions superimposed on the diurnal rhythm. **(E)** Quantification of per-animal mean SPD for different portions of the 24-h cycle. Each dot is one animal, blue circles = male animals; orange = female. Numbers in parentheses indicate hours used for each period. Statistically significant differences are represented by asterisks.

To formally characterize the rhythmic structure of baseline activity, we fit the pooled hourly activity profile under 300 lux with single-component (24-h) and bimodal (24-h + 12-h harmonic) Cosinor models (Figure 3D). Activity was significantly rhythmic, with the dominant 24-h component peaking during the light phase (acrophase ZT9.3), confirming diurnal activity. Adding a 12-h harmonic significantly improved the fit over the single-component model (nested *F*-test, *p* = 0.0004), indicating that baseline activity contains a significant crepuscular (12-h) component superimposed on the dominant diurnal rhythm, consistent with the bimodal activity peaks near the light transitions characteristic of Arvicanthis grass rats (Costello et al., 2023; Caputo et al., 2022; Caputo et al., 2025).

We then quantified SPD means in different daily time windows (Figure 3E). This testing indicates significantly increased activity during the light phase (ZT 0–11) vs. dark phase (ZT 12–23) in the final 2 weeks of the baseline recording (paired *t*-test, *p* = 0.038). Due to significant increases in SPD during the transition periods surrounding the switch from lights ON to OFF (ZT 11–12) compared to the full day (two-tailed paired *t*-test, *p* = 0.0001) and from lights OFF to ON (ZT 23–0) compared to full night (two-tailed paired *t*-test, *p* = 0.008), as a more accurate representation of diurnality (as opposed to crepuscularity), we also compared the middle of the lights ON phase (ZT 3–9) to the middle of lights OFF during the final 2 weeks in 300 lux and saw significant increases in mid-day activity compared to mid-night (ZT 15–21; two-tailed paired *t*-test, *p* = 0.001). Weekly averages across 5 weeks of baseline recordings (including the initial one-week habituation period) showed significant differences in both full day vs. full night as well as mid-day vs. mid-night animal activity for each of the 5 weeks (Supplementary Figure S2). These results indicate that NGRs display strong diurnal cycles of activity across many consecutive days of recording, in our laboratory vivarium environment.

### 3.2 Effect of light alteration on daily movement patterns

Given the influence of light on mood in humans who are diurnal and since NGRs display diurnal activity patterns, we next tested the effects of increased daytime lighting on NGR actigraphy. Environmental lighting is commonly categorized into indoor versus outdoors based on a 1,000 lux light intensity threshold (Read et al., 2014; Ostrin, 2017; Ulaganathan et al., 2019), where the intensity of light below 1,000 lux is generally experienced indoors. Following the baseline period, all 12 animals were exposed to 1,000–1,300 lux light during the day for an additional 4 weeks. A portion of the animals (Cohorts 2–3; *n* = 8) were also then exposed to 4 weeks of constant darkness (DD, 24 h 0 lux) lighting after the 1,000 lux condition (Figure 1).

To assess behavioral activity pattern changes in response to light across the course of the day, we began by plotting mean hourly activity for all animals (*n* = 12 male and female pooled) in the 300 and 1,000 lux LD conditions, using the final 14 days in each condition. Hour-by-hour comparison of the light phases showed higher activity under 1,000 lux across most daylight hours, with the largest and most consistent differences in the early-to-mid light phase (ZT1–8, ZT10; two-tailed *t*-tests, *p* < 0.05 uncorrected; Figure 4A). Although individual hourly differences did not survive Benjamini-Hochberg false-discovery-rate correction across the 24-h scan, the light-phase increase was robust when aggregated: comparing 12-h averages of lights ON and OFF for each condition, bright light significantly increased daytime NGR activity relative to 300 lux-ON (two-tailed paired *t*-test, *p* = 0.021; Figure 4D), though the crepuscular-like rise just prior to the lights-OFF transition in 300 lux mitigates some of this effect. Bright light did not significantly affect activity during the lights-OFF phase (1000-OFF vs. 300 lux-OFF 12-h averages, *p* = 0.59; Figure 4D). Relatedly, in 1000 lux not only did animals expectedly maintain diurnality (1000-ON vs. 1,000-OFF 12-h averages, *p* = 0.002; Figure 4D) but showed a significant increase in diurnality index (DI) relative to 300 lux (paired *t*-test, DI 1000 vs. 300 lux, *p* = 0.003; Figure 4F), driven largely by increased daytime activity. This was a major test of our primary hypothesis.

**Figure 4:**
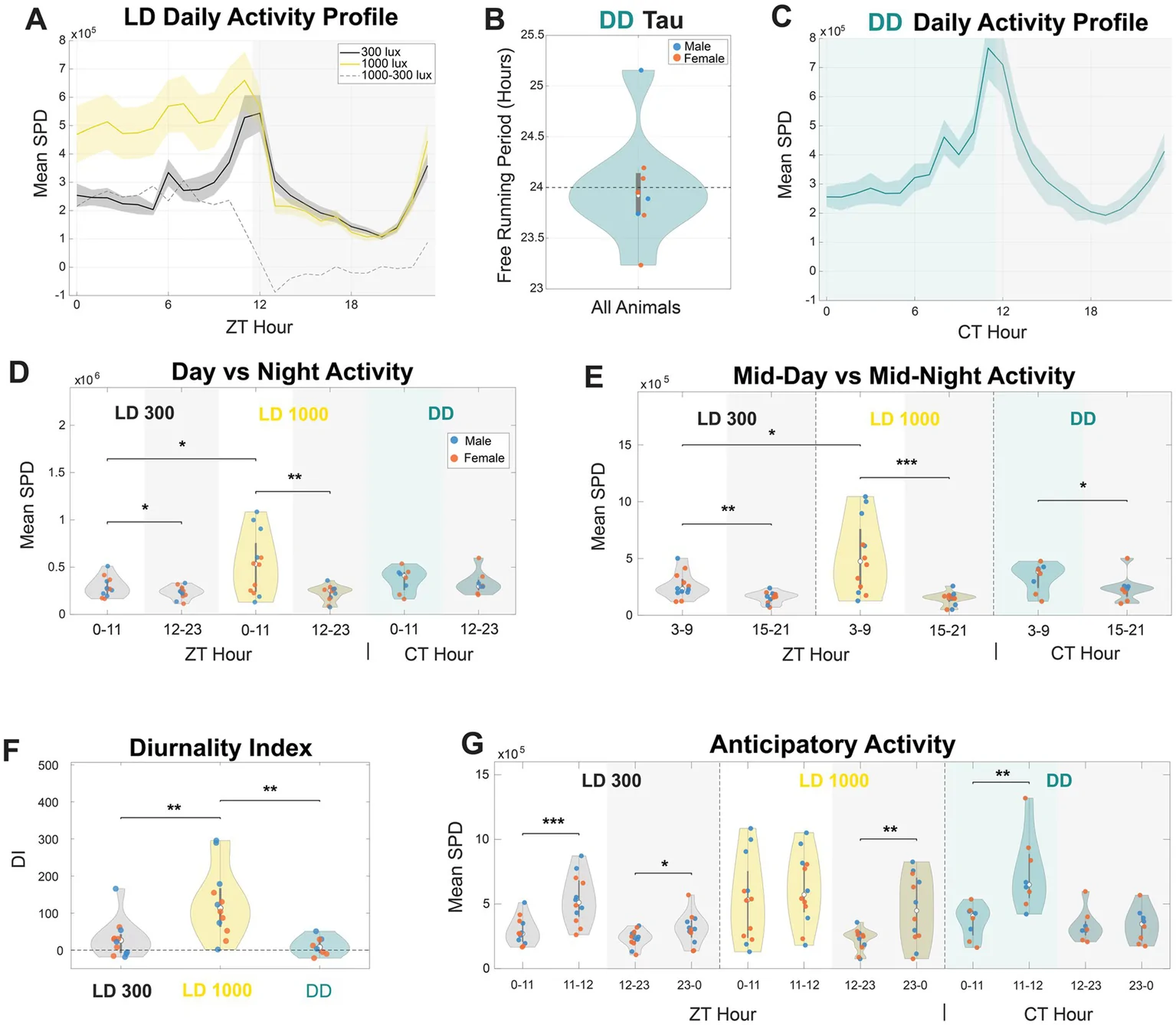
Bright light increases daytime activity and increases diurnality. **(A)** Line plots of hourly averages of *SPD* for 300 lux and 1,000 lux conditions. Dotted line indicates change from 300 lux to 1,000 lux (via subtraction of hourly means). At the uncorrected level, hourly means differed between conditions across most daylight hours (ZT1–8, ZT10; *p* < 0.05), but no individual hour survived Benjamini-Hochberg false-discovery-rate correction across the 24-h scan; the light-phase increase is instead supported by the aggregated analyses in **(D–F)**. The increases in brighter light are concentrated in the light hours, while dark-phase activity is not significantly changed. **(B)** Free running period length (*Tau*) for the last 7 days of all animals that were tested in constant darkness (*n* = 8) as computed by a Lomb-Scargle Fourier transform. Dashed line indicates the expected 24 h rhythm. Male and female individual free running periods represented by blue and orange dots, respectively. **(C)** Constant darkness activity profile for all animals (*n* = 8) in the last 7 days of the constant darkness condition. Each individual animal’s hourly means were re-aligned to CT time (based on the mid-day activity peak) prior to averaging across animals. Pooled hourly means are represented by teal line with teal shading (SEM). **(D)** Quantification of 12-h lights ON and OFF averages for *SPD* by light condition (ZT 0–11 lights ON and ZT 12–23 lights OFF for the 300 lux and 1,000 lux conditions, CT 0–11 for subjective day in constant dark and CT 12–23 for subjective night in constant darkness). Male (blue) and female (orange) data is pooled per violin, and statistically significant differences between groups are represented by asterisks. Differences between day and night in both 300 and 1,000 lux, but difference with 1,000 lux is greater. **(E)** Same as in **(D)** except analysis window is confined to the middle 6 hours of each phase (ZT 3–9 for lights ON, ZT 15–21 for lights OFF; DD CT 3–9 for subjective day and CT 15–21 for subjective night). These results are qualitatively similar to full ON/OFF period analysis except DD condition now also shows a significant difference between subjective day and night (*p* = 0.016). **(F)** Diurnality index (*DI* = (mid-light *SPD* – mid-dark *SPD*)/(mid-dark *SPD*) ×100) comparison for pooled sexes by light condition. **(G)** Quantification of anticipatory peaks of activity during light-transition times (last hour before transition and first hour after transition, ZT/CT 11–12 and ZT/CT 23–0) compared to averages of 12-h lights ON or OFF times (ZT/CT 0–11 and ZT/CT 12–23). Under 300 lux, animals show significant anticipatory activity at both the light-to-dark (dusk) and dark-to-light (dawn) transitions. With 1,000 lux the dusk increase is absent as daytime activity rises to match it, while dawn anticipation persists; and in constant darkness the dusk increase remains while dawn anticipation is absent.

To reduce crepuscular activity contributions in our quantifications, we tested mid-day hours vs. mid-night hours and still saw a significant increase in daytime activity compared to nighttime in the 1,000 lux condition (paired *t*-test mid-1000-ON vs. mid-1000-OFF: *p* = 0.0009, *N* = 12; Figure 4E). We also examined the crepuscular time windows by quantifying differences in anticipatory activity during the final hour of lights ON through the first hour of lights OFF (ZT 11–12) with the full lights ON average (ZT 0–11) for each lighting condition. We found that in 300 lux there is a significant increase in activity just prior to lights OFF (paired *t*-test, 300-ON vs. Dusk: *p* = 0.0001 (*N* = 12); Figure 4G), whereas in the bright light the anticipatory movement prior to lights OFF is not statistically different from the full lights ON period (1000-ON vs. Dusk: *p* = 0.107 (*N* = 12); Figure 4G). Thus, the statistical findings seem to reflect the visual finding of large increases in activity during the mid-ON period with 1,000 lux light while the crepuscular activity values are unchanged between 300 and 1,000 lux (paired *t*-test, 300-Dusk vs. 1,000-Dusk: *p* = 0.45 (*N* = 12); Figure 4G). These findings indicate that brighter daytime lighting increases diurnality in NGRs due to increased activity during the lights ON phase.

Exposing the animals to the DD condition allowed us to observe their circadian pattern and calculate each animal’s free running period during the fourth week of DD using a Lomb-Scargle periodogram (Table 2). The average free running period is 24.0 h, not significantly different from 24 h (one-sample *t*-test, *n* = 8, *p* = 0.99; Figure 4B). However, because of the individual differences in free running period, some shorter some longer than 24 h, we used only the final 7 days of this lighting condition for quantification because using longer periods would incorporate larger shifts in subjective time of day. To account for individual circadian phase difference following several weeks of free running in DD, we aligned each animal’s daily activity profile by circadian time (CT). To do this, we computed each animal’s daily activity profile by averaging hourly activity across the last 7 days of the DD condition, applying a 3-h circular moving average, and identifying the peak activity hour. We found that for each of the 8 animals in the DD environment, the daily activity profile displayed two prominent peaks of activity, directly mirroring the animals entrained by light, only shifted in time (Supplementary Figure S2). Because light-entrained animals in our study all showed the most prominent peak of activity at ZT11 (Figure 4A), we re-aligned each DD animal’s daily activity profile by applying an integer-hour phase offset to shift each animal’s most prominent peak to CT11 (Supplementary Figure S3) prior to pooling and averaging the data for quantification (Figure 4C). This re-alignment to circadian time allows us to quantify each animal’s activity during the subjective day/night despite the individual variations in perceived time of day.

**Table 2:** Free-running period (tau) during fourth week of DD by animal.

Animal ID	Sex	Cohort	Tau
AO5	F	2	23.23
AO6	F	2	24.19
AO7	M	2	23.74
AO8	F	2	23.73
AO9	M	3	25.16
AO10	F	3	23.95
AO11	F	3	24.09
AO12	M	3	23.89

Interestingly, animals in the fourth week of constant darkness still showed significantly more activity during the middle of the subjective lights ON period compared to the middle of the subjective lights OFF period (paired *t*-test, ZT3-9 vs. ZT15-21, *p* = 0.016; Figure 4E) despite the lack of light entrainment. Further, all DD animals still showed anticipatory increases in activity (paired *t*-test, DD-ON vs. Dusk, *p* = 0.003; Figures 4C,G), indicating that these animals naturally follow a diurnal pattern of activity with expectation of light transition. Due to re-alignment of daily activity based on the larger peak at CT11, the anticipatory peak amplitude obscured the 12-h comparison of subjective day vs. night (paired *t*-test, DD-ON vs. DD-OFF, *p* = 0.224; Figure 4D). These animals also showed a small decrease in diurnality index as compared to the 300 lux conditions, though not significant (two-tailed independent *t*-tests, 300 lux DI vs. DD DI, *p* = 0.17; Figure 4F), further providing evidence of an intrinsic diurnal circadian pattern of locomotor activity in the absence of external timing cues.

To formally characterize the circadian structure of activity and how it was modulated by lighting condition, we fit Cosinor models at both the group and individual-animal levels (Figure 5). At the group level, the pooled hourly activity profile for each condition was fit with a single-component (24-h) and a bimodal (24-h + 12-h harmonic) model. All three conditions showed significant 24-h rhythmicity, with the dominant component peaking during the light or subjective-light phase (single-component acrophase: 300 lux, ZT9.3; 1,000 lux, ZT6.4; constant darkness, CT10.4), confirming a fundamentally diurnal organization of activity that persisted even in the absence of light entrainment (Figure 5A). Additionally, in every condition, adding a 12-h harmonic significantly improved the fit over the single-component model (nested *F*-test: 300 lux, *p* = 0.0004; 1,000 lux, *p* = 0.008; constant darkness, *p* < 0.0001), indicating a significant crepuscular (12-h) component superimposed on the dominant diurnal rhythm, consistent with the bimodal activity pattern characteristic of Nile grass rats (Costello et al., 2023; Caputo et al., 2022; Caputo et al., 2025).

**Figure 5:**
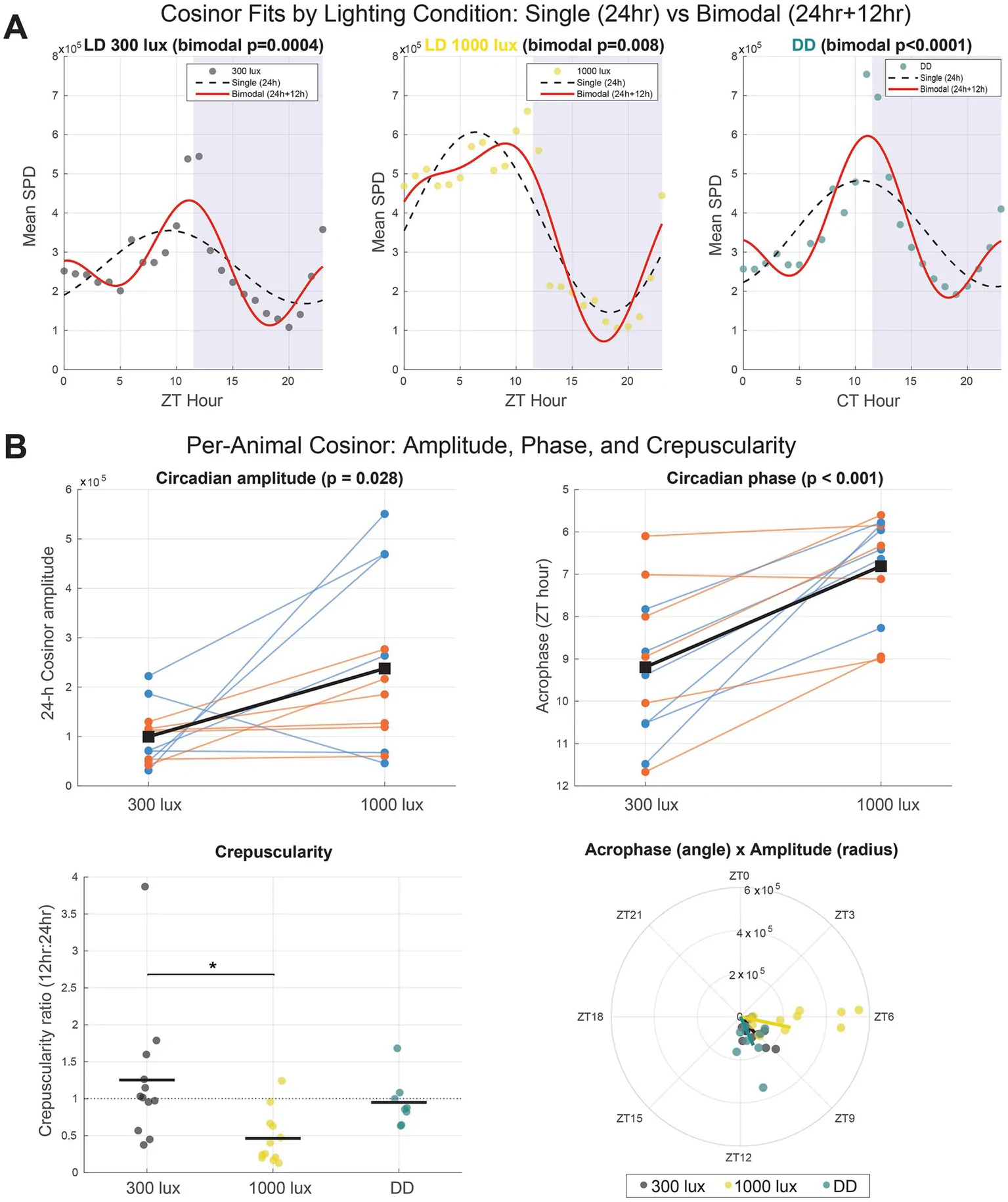
Cosinor analysis reveals significant circadian rhythmicity that is modulated by lighting condition. **(A)** Group-level Cosinor fits of the pooled mean hourly activity profile (selected pixel difference, SPD) for each lighting condition. Each panel shows the observed hourly means (filled circles) overlaid with a single-component (24-h; black dashed) and a bimodal (24-h + 12-h harmonic; red solid) Cosinor model. Time is expressed as ZT for the 300 lux and 1,000 lux conditions and as CT for constant darkness (DD); grey shading indicates the dark phase (lights off) or subjective night. In all three conditions the bimodal model significantly improved the fit over the single-component model (nested *F*-test: 300 lux, *p* = 0.0004; 1,000 lux, *p* = 0.008; DD, *p* < 0.0001), indicating that activity contains a significant crepuscular (12-h) component superimposed on the dominant 24-h diurnal rhythm demonstrated in Figure 4. **(B)** Per-animal Cosinor parameters. Each animal’s mean hourly profile was fit individually per condition. Upper left: 24-h circadian amplitude increased significantly from 300 to 1,000 lux (paired *t*-test, *p* = 0.028; lines connect individual animals, blue = male, orange = female, black = group mean). Upper right: he acrophase of the 24-h component advanced under bright light, peaking approximately 2.4 h earlier at 1000 lux than at 300 lux (paired *t*-test, *p* < 0.001; y-axis inverted so that earlier phase appears higher). Lower left: he crepuscularity ratio (12-h:24-h amplitude) decreased significantly under bright light (paired *t*-test, 300 vs. 1,000 lux, *p* = 0.019; horizontal bars indicate condition means, dotted line marks a ratio of 1). Lower right: olar representation of each animal’s acrophase (angle, ZT/CT hour) and 24-h amplitude (radius), colored by condition, with condition mean vectors shown as radial lines. Note, despite the population findings, individual variation can be strong.

We next fit each animal’s activity profile individually to quantify circadian amplitude, acrophase, and a crepuscularity index (ratio of 12-h to 24-h amplitude) for each condition (Figure 5B). Bright light significantly increased the 24-h circadian amplitude relative to standard lighting (300 lux: 99,631 ± 58,937; 1,000 lux: 237,547 ± 174,222; paired *t*-test, *p* = 0.028) and advanced the acrophase of the dominant diurnal component by approximately 2.4 h (ZT9.2 ± 1.8 vs. ZT6.8 ± 1.3; *p* < 0.001), indicating that brighter daytime light both strengthened and phase-advanced the diurnal activity rhythm. The crepuscularity index decreased significantly under bright light (1.25 ± 0.93 vs. 0.46 ± 0.35; *p* = 0.019); apparently related to the fact that the 12-h amplitude itself did not differ significantly between conditions (*p* = 0.35). This shift reflects the growth of the dominant 24-h component rather than a loss of crepuscular activity, apparently consistent with bright light elevating mid-day activity toward the level of the crepuscular peaks. Interdaily stability (IS), a nonparametric measure of the day-to-day reproducibility of the 24-h activity pattern, was moderate in all conditions and did not differ significantly between 300 and 1,000 lux (0.38 ± 0.15 vs. 0.50 ± 0.21; *p* = 0.17). Per-animal Cosinor parameters and IS values are provided in Supplementary Table 2.

Collectively, these results suggest that NGRs maintain a significant diurnal rhythm across various lighting conditions, and that exposure to bright light increases daytime activity as well as measures of diurnality.

### 3.3 Sex differences and individual variation in response to altered lighting

We next performed exploratory follow-up testing to assess for sex differences in behavioral response to light alterations. Visual comparison of daily averages by sex between 300 and 1,000 lux activity (Figure 6A) reveals apparent differences in how males and females modify their activity profiles when exposed to bright light. Further, after plotting lights ON or OFF means by day and sex, males appear to show sustained increases in activity in the 1,000-ON phase, whereas the females show only small increases in activity in 1000-ON days and small decreases in activity during 1,000-OFF portion of bright days (Figure 6B). To analyze this, we compared group daily means (mean activity across all animals of each sex for each day of recording) and show that males had significantly higher 1,000 lux daytime activity than females (640,040 ± 61,094 vs. 389,292 ± 71,679 pixels/day; *t*(57) = 14.48, *p* < 0.001; Figure 6C). To further compare daily activity levels across all days of the experiment between lighting conditions, we used a Linear Mixed Effects (LME) model (see methods for details). This approach is well suited for our data because each animal contributes many daily observations over an unequal number of recording days; the model uses all daily measurements while accounting for the repeated-measures structure and for stable between-animal differences in baseline activity through a per-animal random intercept, which a comparison of animal means or of pooled days would not capture (see Methods for details). Based on this testing, both males and females significantly increased their daytime 1,000 lux activity levels compared to 300 lux, (males: *β* = 327,875 pixels, *t* = 13.50, *p* < 0.001; females: *β* = 63,482, *t* = 4.3, *p* < 0.001; Figure 6D). No significant effect of lighting condition was observed for male nighttime activity (*β* = −7,770, *t* = −0.74, *p* = 0.46; Figure 6D), whereas females show significantly suppressed nighttime activity (*β* = −39,044, *t* = −4.4, *p* < 0.001; Figure 6D).

**Figure 6:**
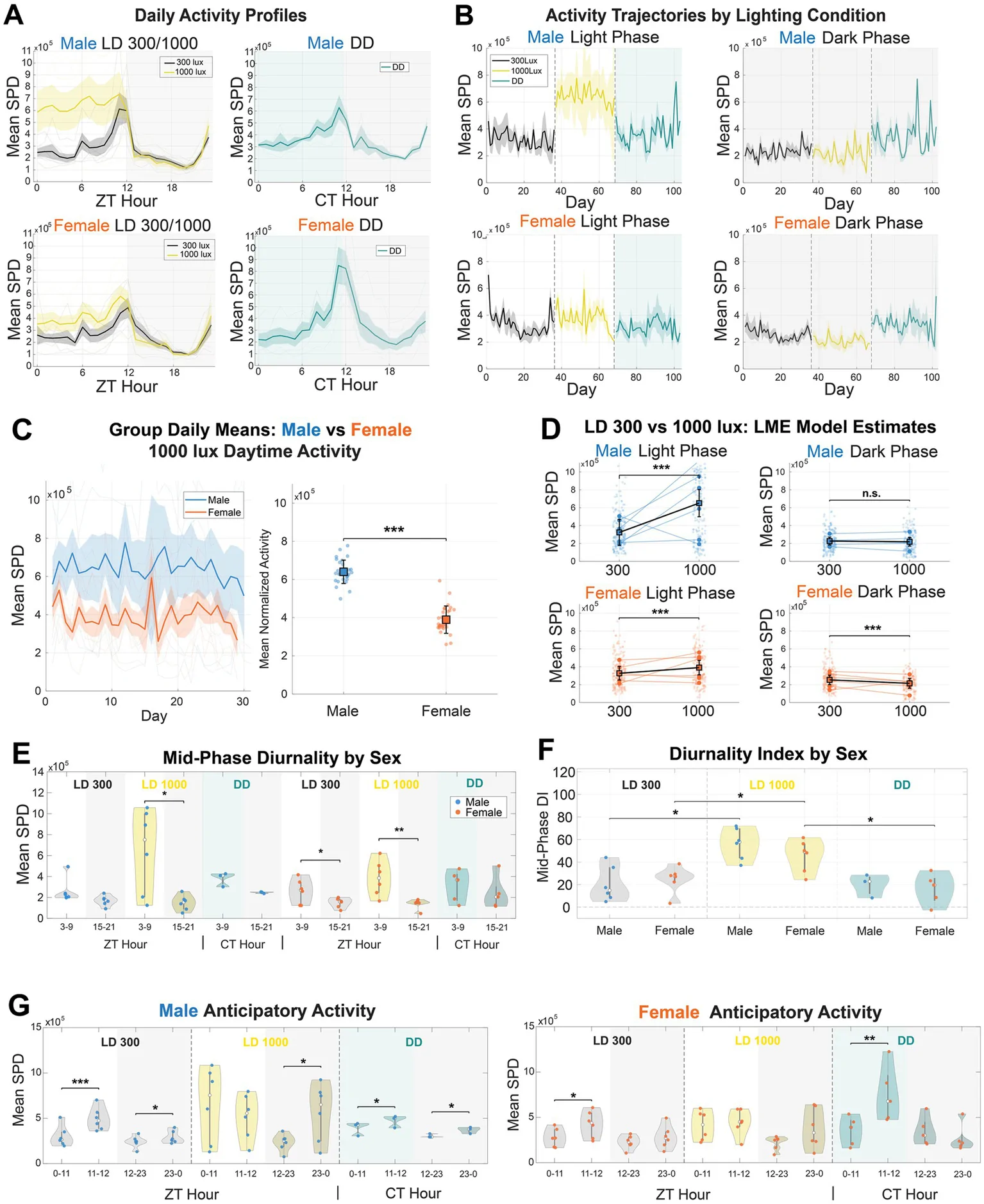
Sex differences in behavioral response to altered lighting conditions. **(A)** Male versus female hourly activity patterns under varying light conditions. Left: Line plots of hourly averages of sex-specific SPD for the LD 300 lux and LD 1000 lux conditions. Male (top) and female (bottom) data plotted separately. At the uncorrected level, hourly means differed between the 300 and 1,000 lux conditions at several daylight hours (males: ZT 1–5, 7; females: ZT 5, 10; *p* < 0.05), but no individual hour survived Benjamini-Hochberg false-discovery-rate correction; sex differences in the light response are supported by the aggregated analyses in **(C,D)**. Right: Constant darkness activity profile for all animals (*n* = 8) in the last 7 days of the DD condition. Each individual animal’s hourly means were re-aligned to CT time prior to averaging across animals by sex. Pooled hourly means are represented by thick line with SEM shading. **(B)** Per-day lights-ON and lights-OFF averages of SPD by sex for all days of each lighting condition. Constant darkness lights ON and OFF use CT times to better represent subjective day/night. **(C)** Left: Subset of data from the 1,000 lux days from **(B)**, overlaid (daily group mean activity) (± SEM across animals) for male (blue) and female (orange) animals across all recording days. Each bold line represents the group mean computed by averaging across all 6 animals of that sex on each day, with shading indicating ± SEM. Faint individual lines show per-animal daily means. Right: Distribution of group daily means for male and female animals. Each point represents 1 day’s group mean (*N* = 30 male days, *N* = 29 female days). Squares at center indicate the mean ± SD across all days. Males showed 64% higher mean daytime activity than females (independent *t*-test, *t*(57) = 14.48, *p* < 0.001). **(D)** Linear mixed-effects model estimates of daytime and nighttime activity at 300 versus 1,000 lux by sex. Faint points show all individual daily activity observations per animal; large squares with error bars show the LME model-estimated condition means ± 95% confidence interval, connected by a bold line. Statistics derive from a mixed-effects model fit to all daily observations with animal as a random intercept, not from a paired comparison of per-animal means. Significance of the condition effect: *** *p* < 0.001, n.s. not significant. Both males and females showed significantly more activity in the lights-ON period in 1000 lux than 300 lux. On the other hand, while 1,000 lux did not change male activity during the lights-OFF, it decreased female activity during lights-OFF. **(E)** Quantification of mid-ON and mid-OFF by light condition and sex to further quantify diurnality across conditions. Male (blue) and female (orange) data is pooled per violin, and statistically significant differences between mid-ON and mid-OFF within each sex condition are represented by asterisks. Note that male 1,000-ON activity is not significantly different than female 1,000-ON, possibly due to the high variability in the male response. Overall, statistical significances are qualitatively similar to the sex-pooled findings in Figure 4E: Both sexes showed the same direction of higher mid-day than mid-night activity across conditions, reaching significance in most conditions, indicating the diurnal pattern is present within each sex. **(F)** Diurnality index across lighting conditions and split by sex (male = blue; female = orange). Note that DI significantly increases in bright light for both sexes. **(G)** Anticipatory activity at the daily light transitions, by sex. Mean activity (selected pixel difference, SPD) in the hour preceding the light-to-dark (dusk) and dark-to-light (dawn) transitions, compared with the corresponding mid-phase baseline (daytime activity for dusk, nighttime activity for dawn), shown separately for males and females across the 300 lux, 1,000 lux, and constant darkness (DD) conditions. Anticipation was tested as a pre-specified primary hypothesis using paired *t*-tests within each sex and condition (reported without multiple-comparisons correction; see Methods and Supplementary Table 1). At dusk, both sexes showed significant anticipatory increases under 300 lux and DD but not under 1,000 lux, where elevated daytime activity matched the late-day peak. At dawn, males showed significant anticipatory increases across all three conditions, including DD (*dz* = −4.24, *p* = 0.018), whereas females showed non-significant increases under 300 and 1,000 lux and no increase under DD. Direct male-versus-female comparisons of pre-dusk and pre-dawn activity showed no significant sex differences in any condition (independent *t*-tests, Benjamini-Hochberg corrected). Data are mean ± SEM; individual animals are shown as points. * *p* < 0.05, ** *p* < 0.01, *** *p* < 0.001; n.s. not significant. *n* = 6 per sex for 300 and 1,000 lux; *n* = 3 males and *n* = 5 females for DD.

We then examined degree of increase from 300 to 1,000 lux. The effect of 1,000 lux light during the daytime was substantially larger in males than females (100% increase in males, 17% increase in females; Figure 6D). At the day level, this sex difference in the light response was captured by a significant sex × condition interaction in the LME model (*β* = 264,331 pixels [95% CI: 208,453–320,210], *t* = 9.287, *p* < 0.001; d = 1.10 [0.87, 1.34]), indicating that males increased daytime activity more than females when exposed to bright light (Supplementary Table 1). However, when the same comparison was made at the animal level, averaging each animal to a single value, the sex difference failed to reach significance (Hedges g = 0.72 [−0.37, 1.80], *n* = 6/sex, *p* = 0.21), likely due to the large variance, particularly in the males (between-animal SD = 271,697 pixels; Figure 6D). This is likely due to the variability of individual responses in the males, with 4 of the 6 males showing increases in activity in 1000-ON (independent *t*-tests, male 1,000-ON vs. male 300-ON, *p* < 0.001 for animal AO1, AO3, AO7, AO9; Supplementary Figure S4; Table 3), while 2 of the males by contrast show no change or a significant decrease in activity (Supplementary Figure S4; Table 3). Females show more modest changes in activity in response to the 1,000-ON condition, with 2 females showing significantly increased activity, 1 significantly decreased activity, and 3 showing no change (Supplementary Figure S4; Table 3).

**Table 3:** 300-ON vs. 1,000-ON activity by sex.

Animal ID	Sex	N_300	300 lux – Mean SPD	N_1,000	1,000 lux – Mean SPD	Delta	t-stat	*p*-value	Significance
AO1	M	35	221,784.4	29	587,536.1	365,751.7	−9.77	<0.001	***Increase
AO2	M	35	210,540.0	31	240,605.9	30,065.9	−1.37	0.175	NS
AO3	M	35	192,106.4	31	821,531.2	629,424.8	−18.75	<0.001	***Increase
AO4	F	35	323,175.2	32	275,867.2	−47,308.0	2.134	0.037	*Decrease
AO5	F	36	393,240.5	26	560,229.4	166,988.9	−5.482	<0.001	***Increase
AO6	F	36	305,080.2	26	298,332.0	−6748.2	0.264	0.792	NS
AO7	M	36	458,196.3	26	950,209.3	492,013.0	−11.67	<0.001	***Increase
AO8	F	36	476,634.0	26	507,894.8	31,260.8	−0.831	0.410	NS
AO9	M	31	364,020.7	29	1,123,896.9	759,876.2	−17.341	<0.001	***Increase
AO10	F	31	212,000.5	29	488,949.9	276,949.4	−7.142	<0.001	***Increase
AO11	F	31	244,764.4	29	219,141.7	−25,622.7	0.639	0.525	NS
AO12	M	31	504,854.2	29	191,988.7	−312,865.5	6.661	<0.001	***Decrease

Animals in constant darkness display individual variation in daily activity, as shown in Figure 4, so we next tested if there were activity differences by sex in that condition. The daily activity profiles are remarkably similar in structure to the activity profiles seen in 300 and 1,000 lux conditions, with two main peaks of activity near expected light transition times even in the absence of light (CT 11 and 23; Figure 6A). This may be due to the fact that even without light entrainment, male and female free running periods remain close to 24 h (male tau, mean = 24.26, std. = 0.78, *N* = 3; female tau, mean = 23.84, std. = 0.38, *N* = 5), and were not significantly different during the final 7 days in constant darkness (independent *t*-test, male vs. female DD tau, *p* = 0.330; Supplementary Figure S5). Further, group daily means testing showed that neither sex had differences across all days of DD-ON daily activity compared to the 300-ON condition (independent *t*-tests, 300-ON vs. DD-ON, male: *t*(61) = − 1.803, *p* = 0.0764, female: *t*(69) = 1.212, *p* = 0.230; Figure 6B), representing a return to baseline activity after coming out of the 1,000 lux condition.

In Figure 4, we showed that diurnality is maintained in various lighting conditions, and that bright light improves diurnality, prior to differentiating by sex. We next tested whether both male and female NGRs show clear diurnal patterns of activity across lighting conditions. Due to anticipatory activity during light transitions, as shown in Figures 4A,G, we excluded light transition times and compared average mid-day (ZT 3–9) and mid-night activity (ZT 15–21). Quantification reveals significantly increased activity during the day compared to night females in both 300 and 1,000 lux conditions and for males in 1000 lux, with males in 300 lux showing a comparable trend that did not reach significance (paired *t*-tests; male 300 mid-ON vs. mid-OFF, *p* = 0.051; male mid-1000-ON vs. mid-OFF, *p* = 0.016; female 300 mid-ON vs. mid-OFF, *p* = 0.018; female mid-1000-ON vs. mid-OFF, *p* = 0.010; Figure 6E). Interestingly, tests for male and female diurnality in the constant dark condition during the final 7 days of each condition showed that neither sex alone had significantly more subjective daytime activity than subjective night activity (paired *t*-tests, male mid-day vs. mid-night, *p* = 0.090; female mid-day vs. mid-night, *p* = 0.148; Figure 6E), though this is likely due to the decrease in testing power when separating by sex (*n* = 3 male, *n* = 5 female), as the pooled sex data showed that animals in constant darkness still follow a diurnal rhythm during the fourth week of DD (Figure 4E). In comparing the sexes during constant dark, we found that males and females show no difference in subjective daytime activity (independent *t*-test, male vs. female DD subjective day, *p* = 0.500; Figure 6E) or subjective night activity (independent *t*-test, male vs. female FD subjective night, *p* = 0.923; Figure 6E), indicating that both sexes show similar activity levels in DD.

Though the maintenance of diurnality was not sex-specific, we next tested whether exposure to altered lighting differentially affects degree of diurnality (DI) in males and females. Both male and female NGRs show significantly increased DI in the bright light conditions (paired *t*-tests, female mid-phase DI 300 vs. female mid-phase DI 1000, *p* = 0.031; male mid-phase DI 300 vs. male mid-phase DI 1000, *p* = 0.034; Figure 6F), but fail to show sex-specific significant differences (independent *t*-tests, male vs. female mid-phase DI, *p* = 0.75 and *p* = 0.16 for 300 and 1,000 lux; Figure 6F). This suggests that both NGR males and females exhibit strong patterns of diurnal activity irrespective of changes to intensity of environmental lighting, and that bright lighting increases diurnality. Interestingly, when combining this finding with the LME in Figure 6D, it may be that males achieve this increased DI in different ways however: males show a robust increase in daytime activity in 1000 lux lighting without modulating night activity, while females show more modest increases in 1000 lux daytime light paired with modest decreases in night activity.

In constant dark, neither males nor females show significant differences in diurnality scores compared to 300 lux (paired *t*-tests, male 300 lux DI vs. DD DI: *p* = 0.202 (*n* = 3); female 300 lux DI vs. DD DI: *p* = 0.430 (*n* = 5); Figure 5F), and there were no sex-specific differences in diurnality index (independent *t*-test, DD-DI male (*n* = 3) vs. female (*n* = 5), *p* = 0.639; Figure 6F). These results suggest that males and females confined to constant darkness maintain a framework of daily activity with a consistent daily running period and anticipatory day/night transition activity that matches their activity level and patterns in 300 lux.

We then investigated whether activity increased prior to the transition into daytime lights-on (dawn) or nighttime lights-off (dusk) by quantifying activity in the hour before each transition. Both males and females show significant increases in activity at dusk when in 300 lux and constant dark conditions (paired *t*-tests, male 300-ON vs. dusk: *p* = 0.0005, male DD-ON vs. dusk: *p* = 0.024, female 300-ON vs. dusk: *p* = 0.041, female DD-ON vs. dusk: *p* = 0.009; Figure 6G), but this anticipatory activity prior to the lights-OFF transition is not different than daytime activity for either sex in 1000 lux. This homogeneity of activity across ON-hours in 1000 lux is not because the dusk activity drops (paired *t*-tests, male 300-dusk vs. male 1,000-dusk: *p* = 0.783, female 300-dusk vs. 1,000-dusk: *p* = 0.303; Figure 6G), but rather an effect of increased daytime 1,000 lux activity that matches the high activity normally only seen during the dusk transition (paired *t*-tests, male 1,000-ON vs. dusk: *p* = 0.863, female 1,000-ON vs. dusk: *p* = 0.078; Figure 6G). For the dark-to-light (dawn) transition, males show significant anticipatory increases in activity across all three lighting conditions (paired *t*-tests, male 300-OFF vs. dawn: *p* = 0.016, male 1,000-OFF vs. dawn: *p* = 0.016, male DD-OFF vs. dawn: *p* = 0.018; Figure 6G), consistent with the crepuscular activity pattern characteristic of NGRs during daily light transitions (Costello et al., 2023; Caputo et al., 2022; Caputo et al., 2025). In females, the pre-dawn comparisons did not reach statistical significance in any condition. In the 300 lux and 1,000 lux conditions the effect was in the same direction as males, reflecting a weak anticipatory increase (dz = −0.69 and −0.88, respectively; 1,000 lux *p* = 0.084), whereas in constant darkness female pre-dawn activity showed no such increase (dz = +0.45, *p* = 0.367). Direct comparison of male and female dawn activity revealed no significant sex differences in any condition (independent *t*-tests, *p* = 0.932 for 300 lux, *p* = 0.380 for 1,000 lux; Figure 6G). These patterns suggest that females exhibit a weaker and less consistent pre-dawn anticipatory response than males, particularly in the absence of a light cue, though this cannot be considered a categorical sex difference given the limited power of the per-sex comparisons and the absence of significant male–female differences.

## 4 Discussion

In this study we use automated monitoring systems to show that in a diurnal rodent, brighter light induces increased daytime-specificity of activity both sexes, but in varying patterns by sex.

The automated monitoring system involves pixel-based actigraphy tracking for single-housed rodents. This tracking system is efficient, able to be automatically processed in batches, and can be seamlessly applied across animals within the same vivarium conditions. Pixel-based actigraphy offers the advantage of continuous recording, higher spatial resolution, more dense sampling, and is more sensitive to pixel gradient changes compared to other tracking methods (i.e., infrared sensors). Many video-based software programs offer similar advantages but are expensive and can be overly complex for basic tracking needs, whereas our tracking system is simple and freely available as open-source Python code. The present study demonstrates the effectiveness of using this system for characterizing in-cage locomotor activity in rodents.

Many rodent models are nocturnal, a trait shaped by evolutionary pressures to avoid predators and exploit nighttime niches. Diurnal rodents like the NGR have evolved adaptations (e.g., visual systems, hormonal rhythms, behavior) for daytime activity. This study helps clarify which aspects of light’s effect on brain and behavior are universal versus those that diverge in diurnal vs. nocturnal species.

Evidence that these animals all show a near 24-h daily rhythm after a month in constant darkness conditions indicates a strong endogenous circadian rhythm—an evolutionary adaptation favoring activity during daylight. The anticipatory activity preceding the dark-to-light transition that persists in constant darkness, most robustly in males, supports the idea that these animals are endogenously primed to increase activity in anticipation of light, independent of an external light cue. In males this pre-dawn increase remained large and statistically significant even in the absence of a light–dark cycle, whereas in females it was weaker and less consistent, appearing more dependent on the presence of a light cue. Though our creation of circadian time by manual re-alignment of daily activity profiles produces artificial summation of peak activity prior to lights OFF, the individual circadian-shifted profiles all show near 12-h separation between distinct peaks of activity, and the secondary peak, which is not forced into alignment, persists after pooling CT profiles across animals. Together, these observations suggest that these animals inherently maintain bimodal daily activity rhythms, with an endogenous anticipatory component that is most pronounced in males.

Our data provide evidence that NGRs display features of both diurnality and crepuscularity under standard vivarium conditions. These animals are clearly diurnal, as shown by the mid-phase analyses that compare activity after removing the transition periods, yet they also exhibit reproducible activity peaks near both daily light transitions. Cosinor analysis confirmed that this bimodal structure is a genuine feature of the activity rhythm: a 12-h harmonic significantly improved the fit in all lighting conditions, including constant darkness (DD), indicating that the crepuscular component is endogenously driven rather than an artifact of standard lighting and consistent with the bimodal activity described in NGRs (Costello et al., 2023; Caputo et al., 2022; Caputo et al., 2025). Under 1,000 lux, this anticipatory crepuscular increase was attenuated (Figures 4G, 5G); rather than eliminating the underlying rhythm, bright light raised daytime activity toward the level of the crepuscular peaks, reducing the relative prominence of the transition-associated activity (as reflected in the reduced crepuscularity index under bright light; Figure 5).

Prior work in NGRs has shown that dim daytime lighting (~50 lux) induces depressive-like behaviors relative to a 1,000 lux condition (Leach et al., 2013; Yan et al., 2019). Our finding that daytime activity increases further under 1,000 lux relative to 300 lux extends this relationship across a broader range of intensities, suggesting that the effect of daytime light on diurnal activity is graded rather than reflecting a single dim-light threshold. We speculate, therefore, that standard vivarium lighting at 300 lux, while clearly more favorable than the ~50 lux conditions previously associated with depressive-like phenotypes, may itself fall below the light intensity that maximizes diurnal activity in this species, and could represent a suboptimal environment relative to the brighter, broad-spectrum light these animals would experience in their natural habitat. This interpretation is speculative: our study did not include measures of mood or depressive-like behavior, and testing whether the activity differences we observe correspond to affective differences would require dedicated behavioral and endocrine assays.

Additionally, rodent research has prioritized the use of male-only data, generally due to the difficulty of controlling for the female estrous cycle, or pooled data of males and females. This approach fails to address the sex-specific differences seen in humans, where females are 1.6–2.0x more likely to be affected by SAD, MDD and anxiety disorders (Altemus et al., 2014; Li et al., 2023; Eid et al., 2019; Kim et al., 2025). Men and women also show a difference in antidepressant efficacy and treatment response for MDD and anxiety disorders (Pavlidi et al., 2022; Silva et al., 2023; LeGates et al., 2019). These observations indicate that independent scientific exploration of the biology of both sexes is essential for translation to human disease and necessitates a closer examination of the underlying differences in neurobiological and hormonal mechanisms.

The neurobiological basis for these sex differences is multifaceted. At the circuit level, males and females differ in the organization and response of limbic and prefrontal regions central to mood regulation, including the hippocampus, amygdala, and medial prefrontal cortex (Bangasser and Cuarenta, 2021; Williams et al., 2022; Mohammadi et al., 2023; Dong et al., 2024). These differences in response are tied to circadian timing of glucocorticoid rhythms, which are themselves under circadian control and differ in amplitude and phase between sexes as well as modulate mood-related circuit excitability in a sex-specific manner (Bangasser and Cuarenta, 2021; Williams et al., 2022). Gonadal hormones add a further layer of complexity: estrogen affects serotonergic and dopaminergic systems that regulate both mood and circadian output (Pavlidi et al., 2022; Silva et al., 2023; LeGates et al., 2019). Notably, NGRs are induced ovulators and do not undergo spontaneous estrous cycling in the absence of a male conspecific. This means that single-housed females in the current study are unlikely to experience the hormonal fluctuations associated with spontaneous cycling seen in other rodent models, which may be an advantage of the NGR model for studying sex differences in light responsiveness, as observed differences between males and females are less likely to be intertwined with cycle-dependent hormonal variation. Testosterone, conversely, has been shown to modulate arousal and locomotor drive in ways that may amplify behavioral responses to environmental stimuli such as light (Bangasser and Cuarenta, 2021).

Evidence from NGR studies has shown that daytime light deficiency can produce depression-like behavioral changes, including reduced sucrose preference and altered social behavior, and importantly, that these responses are sex specific. Low daytime light intensity induced sex and brain region-specific neuroinflammatory responses in NGRs, suggesting that the neural circuits mediating light sensitivity are organized differently across sexes even within a diurnal species (Costello et al., 2023). Also, lesion studies targeting the dorsal raphe nucleus, a major serotonergic hub involved in both mood regulation and circadian control, demonstrate that this region exerts control over daytime locomotor activity in NGRs in a sex-specific manner, and the effects are independent of the estrous cycle (Tamogami et al., 2025). Taken together, these findings from diurnal rodent models provide strong motivation for examining sex as a biological variable in studies of light-driven circadian behavior and suggest that observed sex differences in light responsiveness are likely to reflect genuine neurobiological divergence rather than individual variability.

Indeed, here we find that exposure to bright light improves diurnality scores for both males and females, though the data suggests that the male response in 1000 lux conditions is much more robust and variable compared to females, whereas females both modestly increase daytime activity and modestly decrease nighttime activity. This sex-specific behavioral modulation is further demonstrated by hourly activity profiles in the 1,000 lux condition which show marked increases in male activity, especially during the first 6 h of daytime light. We speculate that the observed sex difference could relate to divergent reproductive and social strategies, whereby females may be more responsive to environmental and social context and males more strongly driven by territorial or mate-seeking behaviors. This interpretation is speculative and not directly tested by our data; distinguishing these possibilities would require designs incorporating reproductive state, social context, and hormonal measures. Recent evidence has shown that lesions in the dorsal raphe nucleus selectively increase female daytime activity irrespective of the estrus cycle, which suggests there are sex-specific differences in how the same brain region exerts circadian control of movement (Tamogami et al., 2025), and offers insight into why males and females respond differently to the 1,000 lux light in our study.

The primary light-condition effects are large (Cohen’s d > 0.8) and correspond to substantial changes in daily behavior. Exposure to bright light increased daytime activity in males by approximately 100% (LME standardized d = 1.12) and in females by approximately 17% (d = 0.38), and significantly increased the mid-phase diurnality index in both sexes. Because the diurnality index reflects the distribution of activity between the light and dark phases rather than overall activity level, these changes indicate a behavioral reorganization, not simply a change in the animals’ movement.

This study was designed as an initial pilot-level characterization of circadian behavioral responses to varying light conditions in NGRs over extended recording periods. Prior actigraphy and behavioral studies in NGRs have detected significant circadian and light-driven effects with comparable sample sizes of 6–10 animals (Leach et al., 2013; Lonstein et al., 2019), and our primary light-condition effects were correspondingly robust in the full cohort of 12 animals. Analyzed at the level of individual recording days, the sex-by-condition interaction was large and statistically significant (mixed-effects model: *β* = 264,331, d = 1.10, *p* < 0.001), with males showing a markedly greater increase in daytime activity under bright light than females (~100% vs. ~ 17%). A more conservative analysis treating each animal as a single observation did not reach significance (Hedges’ g = 0.72, *n* = 6 per sex, *p* = 0.21).

The direction and magnitude of these analyses appear to indicate a sex difference in the behavioral response to light, whereas the difference in statistical significance reflects the limited number of biological replicates rather than the absence of an effect. A simulation-based power analysis indicated that, whereas the cohort was well powered for the primary light-condition effects, it had only ~25% power to resolve the sex difference at the animal level, where approximately 28 animals per sex would be required for 80% power. Because sex differences were a secondary, exploratory aim, the present study is underpowered to characterize them definitively at the animal level, and the magnitude of the sex effect and physiological correlates should be confirmed in a cohort designed and powered specifically for that purpose.

It’s also possible that the high variability in the male response to 1,000 lux light, compared to females, is a result of the artificial lighting in an unnatural vivarium setting. In this study we followed the typically used >1,000 lux threshold for separating indoor and outdoor lighting, with indoor levels generally measuring in the range of 100–500 lux (Daugaard et al., 2019). Our bright light condition may not have been sufficient intensity for all males to alter their activity, considering that outdoor light intensities can range from 1,000 to 100,000 + lux depending on time of day, cloud cover, and environmental surroundings (Norton and Siegwart, 2013; Lou and Ostrin, 2023; Lanca et al., 2019), though the experienced peak intensities generally measure less than 15,000 lux for the brightest days due to lens filtering properties, and average closer to 1,000–5,000 lux depending on other environmental conditions (Lanca et al., 2019). Further, most commercially available LED lights are restricted in light emission to certain wavelengths, with many of the missing wavelengths having high relevance for grass rats (Kim et al., 2023), unlike natural sunlight that encompasses a broad spectrum of wavelengths. It is also unknown if male and female NGRs are differentially sensitive to light, thus, future studies using higher intensity lighting or covering a broader spectrum of wavelengths may better discriminate sex differences in response to bright light.

There is also a possibility that sex-specific responses to 1,000 lux light exposure (male individual variability, female attenuation compared to male, and robustness of male activity) are due to stress, lack of social interaction, or depression, which themselves may differentially affect males and females. For example, female daily activity averages over the months-long experiment indicates that females generally decrease activity in the lights-ON phase over time, both from day 0 versus 90 and first vs. last day of each condition (Figure 5B). A new design with lighting condition order reversed would be needed to test this, along with measures for stress hormones and other behavioral indicators of depression. Further, the highly variable male response to the 1,000 lux lighting could also indicate individual behavioral phenotypes (i.e., responders and non-responders) often seen with therapeutic intervention for depression, such as antidepressants and light therapy. The more robust activity increase under bright light in more males than females is consistent with, but does not by itself demonstrate, a sex-specific adaptive response; this remains a hypothesis for future investigation.

Although the present study was behavioral, our findings may be attributable to neurobiological circuitry known to mediate light’s effects on activity and mood in this diurnal model. The circadian organization we observed, including the near-24-h free-running rhythm in constant darkness, is consistent with a strong endogenous pacemaker in the suprachiasmatic nucleus (SCN). Further, in other species of grass rat (*Arvicanthis ansorgei*), behavioral activity has been shown to shape SCN electrical activity, which may indicate that the activity patterns we observed could be integrated at the level of the clock (Caputo et al., 2025). The crepuscular rhythm of activity we quantified also parallels the rhythmic serotonergic signaling seen in grass rats, where tryptophan hydroxylase and 5-HT rhythms peak near the light transitions while melatonin rhythms follow the day-night cycle (Caputo et al., 2022). Serotonergic circuits are further implicated in the sex differences we observed, as lesions of the dorsal raphe nucleus selectively increase daytime activity in female grass rats (Tamogami et al., 2025). Importantly, the same daytime-light manipulations that alter activity in this model have also been shown to affect emotional, cognitive, and stress-related outcomes: dim daytime light induces depressive and anxiety-like behaviors (Leach et al., 2013), impairs hippocampal function and spatial learning (Soler et al., 2018; Soler et al., 2019), and elicits sex and brain region-specific neuroinflammatory responses (Costello et al., 2023). This convergence suggests that the activity changes we report are unlikely to occur in isolation and may demonstrate broader effects of daytime light on affect, cognition, and stress physiology, mediated through overlapping circadian and limbic circuitry. Establishing these links directly will require studies that pair activity monitoring with behavioral assays of emotion and cognition, measures of stress hormones, and targeted recordings from the underlying circuits.

Several potential confounding variables were considered in the design and interpretation of the current study. Cage position within the vivarium rack was controlled by randomly assigning animals to positions on the top rack of the vivarium shelving, where light intensity was consistently measured at 300 lux under standard conditions. All animals within a cohort were therefore exposed to equivalent light intensities, and overhead LED supplementation during the 1,000 lux condition was applied uniformly across all cage positions. Noise and temperature were maintained at standard vivarium levels throughout the study and did not vary systematically between conditions or cohorts. Habituation to the recording environment was addressed by beginning data collection only after a minimum of 1 week of single housing acclimation, and by excluding the first 7 days of each lighting condition from the analyses, allowing animals sufficient time to acclimate to each new condition prior to quantification.

Several limitations should be considered when interpreting these findings. First, as discussed above, the study was powered for the primary light-condition effects but underpowered to characterize sex differences definitively at the animal level; the sex effect should be confirmed in a larger cohort designed for that purpose. Second, our measurements were restricted to locomotor activity: we did not collect physiological biomarkers, hormonal measurements, or assessments of affective state. As a result, although our activity data are consistent with prior work linking daytime light intensity to mood-related phenotypes in this model, we cannot directly link the activity changes we observe to underlying physiological, endocrine, or emotional processes. In particular, the absence of corticosterone or other stress-hormone measures limits our ability to distinguish lighting effects from possible cumulative effects of single housing, and the absence of gonadal hormone measures limits interpretation of the sex differences; measures such as corticosterone, gonadal hormones, and validated behavioral assays of depressive- and anxiety-like behavior would be required to test the interpretations proposed here. Finally, the fixed order of lighting conditions introduces a potential order effect that cannot be fully disentangled from the lighting effects in the present design, and a counterbalanced or Latin-square design would be needed to control for order, habituation, and cumulative stress.

We also note that single housing, while standard practice for actigraphy studies to prevent animal interactions from confounding the movement signal, can itself induce stress-related behavioral changes in rodents. The gradual decline in female activity over the course of the experiment (Figure 6B) may in part reflect cumulative effects of prolonged single housing rather than lighting condition per se, a possibility that the fixed order of conditions in our design cannot fully exclude. Future actigraphy-focused studies that do not require single housing for physiological recordings could employ RFID-assisted tracking in group-housed animals to disentangle isolation effects from lighting effects.

These findings have potential relevance for understanding light-based interventions in humans, particularly for Seasonal Affective Disorder (SAD) and other conditions treated with bright-light therapy. This is because like the diurnal grass rat, humans are a day-active species whose mood and circadian physiology are sensitive to daytime light intensity, and bright-light therapy is a first-line treatment for SAD (Burgess et al., 2004; Terman and Terman, 2005). Our observation that increasing daytime light intensity from standard indoor levels (300 lux) to brighter light (1,000 lux) increases daytime movement and diurnal activity is consistent with the premise underlying clinical light therapy: that ambient indoor lighting may be insufficient to fully support diurnal physiology, and that supplemental bright light can shift behavior toward a stronger day-active pattern. The marked individual variability we observed, particularly among males, also parallels the well-documented heterogeneity in human responses to light therapy, in which some individuals respond strongly and others minimally (Terman et al., 1996). Characterizing the biological basis of such responder and non-responder phenotypes in a diurnal animal model could inform efforts to predict and personalize light-based treatment; however, these connections should be drawn with caution given the differences between NGRs and humans (i.e., visual system properties) and that our activity-based measures do not directly assess animal mood. Several lines of future research may follow from this work. First, because commercially available LED lighting, as used here, is spectrally restricted relative to natural sunlight, and because the wavelengths it omits may be behaviorally relevant to grass rats, future studies should manipulate spectral composition as well as intensity, testing broader-spectrum and higher-intensity light to determine whether the sex differences and individual variability we observed are sharpened or attenuated under more naturalistic lighting. Along similar lines, incorporating gradual light transitions rather than abrupt light–dark switches would more closely approximate natural conditions, and examining how transition profile interacts with the crepuscular activity of this species is a further direction worth pursuing. Second, pairing activity monitoring with endocrine measurements, including corticosterone and gonadal hormones, would help distinguish lighting effects from stress and reproductive influences and clarify the physiological basis of the sex-specific responses. Third, incorporating behavioral assays for depression and anxiety would allow the activity changes we observe to be linked directly to mood disorders and therapeutic interventions. Fourth, neuroimaging and *in vivo* neurophysiological approaches could identify the circadian and limbic circuits, including the SCN, dorsal raphe, and associated mood-regulating regions, through which daytime light intensity shapes activity and affect. Finally, a counterbalanced design incorporating these measures in a larger, better-powered cohort, informed by the effect-size estimates provided here, would enable a definitive characterization of sex differences and their neurobiological correlates.

Taken collectively, this work emphasizes the importance of further investigating potential sex differences in response to light especially in models sharing diurnality with humans to better understand and treat circadian-associated neuropsychiatric disorders.

## 5 Conclusion

Using automated, open-source pixel-based actigraphy, we show that increasing daytime light intensity increases daytime movement and strengthens diurnal activity in the Nile grass rat, a diurnal rodent, and that this response is sex-dependent, with males showing a larger and more variable increase than females. Methodologically, this work demonstrates that continuous pixel-based actigraphy can resolve circadian amplitude, phase, and crepuscular structure, as well as individual variability, across months-long recordings. For human application, the light-varying, sex-dependent and highly individual responses we observed point to two translational priorities: understanding why individuals differ in their behavioral response to light, and whether these differences are sex-specific, both of which may affect the personalization of light therapy and the design of daytime lighting environments. Definitively establishing these sex-based differences, and the neurophysiological mechanisms that underlie them, will require appropriately powered and counterbalanced studies that pair activity monitoring with direct physiological, endocrine, and neural measurements.

## Data Availability

The data analyzed in this study are available from the corresponding author upon request. The analysis code supporting this study, including the master actigraphy output file and the MATLAB scripts used to process the data and generate all figures, are publicly available in a GitHub repository: https://github.com/BrendonWatsonLab/ActigraphyWork/tree/main/ActigraphyAnalysis.

## Footnotes

1
https://github.com/BrendonWatsonLab/ActigraphyWork/tree/main/ActigraphyCode
