# Supplementary material for: Daytime lighting conditions alter home-cage behavior across both sexes in diurnal grass rats
Source: Front Neurosci. 2026 Sep 17;20:1882564. doi: 10.3389/fnins.2026.1882564 (PMC13627377; doi:10.3389/fnins.2026.1882564)
Supplement: Supplementary file 1 [file Data_sheet_1.docx]

Supplementary Material

# 1 Supplementary Figures

**Supplementary Figure 1. Correlation between SPD and Euclidean distance metric for calculating animal activity in the cage in 1h bins.**

Correlation between total SPD and total XY distance (Summed Euclidean distance) for 1h bins in one animal over multiple days. Total SPD was the result of the pixel tracking output algorithm, while Euclidean distance was quantified by manually labeling the center mass of the animal and calculating the change in XY distance over time. Resulting data showed high correlation between pixel tracking and Euclidean distance (Pearson’s r=0.95), indicating the actigraphy method reliably tracked animal movement.

**Supplementary Figure 2. Weekly diurnality testing in standard vivarium lighting.** Quantification of weekly mean normalized SPD for comparison of different portions of the day (blue circles = male animals; orange = female). Each plot represents a different week in the 300 lux baseline condition. Weekly separation demonstrates stability of diurnality in the baseline condition.

**Supplementary Figure 3. Daily activity profiles prior to re-alignment for Circadian Time.** Creation of a new circadian time (CT) was performed by first plotting daily activity profiles for each animal composed of hourly averages across ZT time for the last 7 days in constant darkness. The black line shows the average SPD by hour and smoothed using a 3-hr moving average. The grey line represents the raw hourly average. The red vertical line indicates the peak activity of each animal and serves as the anchoring point for re-alignment of each animal’s hourly data by defining the peak as the new CT 11 (blue dashed line) to match the ZT 11 peak seen in both male and female data in light-entrained conditions (Fig. 3-5).

**Supplementary Figure 4. Individual variability in male activity following exposure to bright lights.** (A) Individual animal daily mean SPD across months-long experiment (top: males, bottom: females). Data is smoothed using a 3-day moving average. Last 14 days was chosen as the analysis time window based on settling of animal movement after the first 2 weeks, particularly in females (grey box). Notice the activity increase in the males in response to the 1000 lux condition as well as the individual variability in the male response compared to female. (B) Quantification of daily averages from the last 14 days of 300 lux and 1000 lux conditions to show the individual trajectories of each male and female in response to the 1000 lux condition. Notice that in lights-OFF both male and female variability is similar, but in lights-ON males show both larger increases and larger decreases in trajectories than the females.

**Supplementary Figure 5. Free running period (tau) by sex.** Free running period (tau) computed using a Lomb-Scargle Fourier transform during the final 7 days of the constant darkness condition by sex (n=3 male, n=5 female). Dashed line indicates the expected 24-hr rhythm. No significant differences are found either between sexes or from the 24-hr daily rhythm; however, notice that some animals experience subjectively longer days and some shorter, forming the basis for the need to re-align daily activity profiles for each animal individually using CT time.

# 2 Supplementary Tables

Supplementary Table 1:

| **Fig.** | **Comparison** | **N** | **t** | **p** | **q (FDR)** | **Effect size** | **95% CI** | **Sig.** |
| --- | --- | --- | --- | --- | --- | --- | --- | --- |
| **Manuscript Fig. 3E (Within-Day Activity, pooled) - planned primary** | | | | | | | | |
| **3E** | Light vs Dark | 12 | 2.342 | 0.0390 | — | **Cohen's d_z = 0.68** | [0.05, 1.30] | ***** |
| **3E** | Mid-Light vs Mid-Dark | 12 | 4.391 | 0.0011 | — | **Cohen's d_z = 1.27** | [0.51, 2.03] | ****** |
| **3E** | Dusk vs Day avg | 12 | 5.939 | 0.0001 | — | **Cohen's d_z = 1.71** | [0.83, 2.60] | ******* |
| **3E** | Dawn vs Night avg | 12 | 3.092 | 0.0102 | — | **Cohen's d_z = 0.89** | [0.22, 1.56] | ***** |
| **Manuscript Fig. 4D (Day vs Night Activity) - planned primary** | | | | | | | | |
| **4D** | 300lux ON vs OFF | 12 | 2.342 | 0.0390 | — | **Cohen's d_z = 0.68** | [0.05, 1.30] | ***** |
| **4D** | 1000lux ON vs OFF | 12 | 3.894 | 0.0025 | — | **Cohen's d_z = 1.12** | [0.40, 1.85] | ****** |
| **4D** | FullDark ON vs OFF | 8 | 1.420 | 0.1985 | — | **Cohen's d_z = 0.50** | [-0.23, 1.24] | **ns** |
| **4D** | 300lux ON vs 1000lux ON | 12 | -2.701 | 0.0206 | — | **Cohen's d_z = -0.78** | [-1.43, -0.13] | ***** |
| **4D** | 300lux OFF vs 1000lux OFF | 12 | 0.559 | 0.5873 | — | **Cohen's d_z = 0.16** | [-0.41, 0.73] | **ns** |
| **4D** | 1000lux ON vs FullDark ON | 8 | 1.357 | 0.2168 | — | **Cohen's d_z = 0.48** | [-0.25, 1.21] | **ns** |
| **Manuscript Fig. 4E (Mid-Day vs Mid-Night Activity) - planned primary** | | | | | | | | |
| **4E** | 300lux MidLight vs MidDark | 12 | 4.391 | 0.0011 | — | **Cohen's d_z = 1.27** | [0.51, 2.03] | ****** |
| **4E** | 1000lux MidLight vs MidDark | 12 | 4.512 | 0.0009 | — | **Cohen's d_z = 1.30** | [0.53, 2.07] | ******* |
| **4E** | DD MidLight vs MidDark | 8 | 3.164 | 0.0158 | — | **Cohen's d_z = 1.12** | [0.24, 2.00] | ***** |
| **4E** | 300lux ON vs 1000lux ON (mid) | 12 | -2.905 | 0.0143 | — | **Cohen's d_z = -0.84** | [-1.50, -0.18] | ***** |
| **Manuscript Fig. 4F (Diurnality Index, mid-phase) - planned primary** | | | | | | | | |
| **4F** | DI 300 vs 1000lux | 12 | -3.716 | 0.0034 | — | **Cohen's d_z = -1.07** | [-1.78, -0.36] | ****** |
| **4F** | DI 1000lux vs DD | 8 | 4.170 | 0.0042 | — | **Cohen's d_z = 1.47** | [0.47, 2.48] | ****** |
| **4F** | DI 300lux vs DD | 8 | 1.523 | 0.1715 | — | **Cohen's d_z = 0.54** | [-0.20, 1.28] | **ns** |
| **Manuscript Fig. 4B (Free-Running Period) - planned primary** | | | | | | | | |
| **4B** | Pooled tau vs 24h | 8 | -0.013 | 0.9901 | — | **Cohen's d = -0.00** | [-0.70, 0.69] | **ns** |
| **Manuscript Fig. 4G (Light-Transition Anticipation) - within-condition Day-vs-Dusk & Night-vs-Dawn planned; between-condition FDR** | | | | | | | | |
| **4G** | 300lux Day vs Dusk | 12 | -5.939 | 0.0001 | — | **Cohen's d_z = -1.71** | [-2.60, -0.83] | ******* |
| **4G** | 300lux Night vs Dawn | 12 | -3.092 | 0.0102 | — | **Cohen's d_z = -0.89** | [-1.56, -0.22] | ***** |
| **4G** | 1000lux Day vs Dusk | 12 | -1.753 | 0.1074 | — | **Cohen's d_z = -0.51** | [-1.11, 0.09] | **ns** |
| **4G** | 1000lux Night vs Dawn | 12 | -4.041 | 0.0019 | — | **Cohen's d_z = -1.17** | [-1.90, -0.43] | ****** |
| **4G** | DD Day vs Dusk | 8 | -4.346 | 0.0034 | — | **Cohen's d_z = -1.54** | [-2.56, -0.51] | ****** |
| **4G** | DD Night vs Dawn | 8 | -0.125 | 0.9039 | — | **Cohen's d_z = -0.04** | [-0.74, 0.65] | **ns** |
| **4G** | Dusk 300 vs 1000 | 12 | -0.790 | 0.4462 | 0.6694 | **Cohen's d_z = -0.23** | [-0.80, 0.35] | **ns** |
| **4G** | Dusk 1000 vs DD | 8 | -0.409 | 0.6945 | 0.7388 | **Cohen's d_z = -0.14** | [-0.84, 0.55] | **ns** |
| **4G** | Dusk 300 vs DD | 8 | -1.120 | 0.2997 | 0.5995 | **Cohen's d_z = -0.40** | [-1.12, 0.32] | **ns** |
| **4G** | Dawn 300 vs 1000 | 12 | -2.705 | 0.0205 | 0.1229 | **Cohen's d_z = -0.78** | [-1.43, -0.13] | **ns** |
| **4G** | Dawn 1000 vs DD | 8 | 1.231 | 0.2581 | 0.5995 | **Cohen's d_z = 0.44** | [-0.29, 1.16] | **ns** |
| **4G** | Dawn 300 vs DD | 8 | -0.347 | 0.7388 | 0.7388 | **Cohen's d_z = -0.12** | [-0.82, 0.57] | **ns** |
| **Manuscript Fig. 6C (Sex difference, animal-level) - secondary** | | | | | | | | |
| **6C** | M vs F 1000lux ON (per-animal) | 6/6 | 1.345 | 0.2084 | — | **Hedges' g = 0.72** | [-0.37, 1.80] | **ns** |
| **Manuscript Fig. 6E (Mid-Phase Diurnality by Sex) - planned primary** | | | | | | | | |
| **6E** | Male 300lux ML vs MD | 6 | 2.559 | 0.0507 | — | **Cohen's d_z = 1.04** | [0.05, 2.04] | **ns** |
| **6E** | Male 1000lux ML vs MD | 6 | 3.551 | 0.0164 | — | **Cohen's d_z = 1.45** | [0.30, 2.60] | ***** |
| **6E** | Female 300lux ML vs MD | 6 | 3.478 | 0.0177 | — | **Cohen's d_z = 1.42** | [0.29, 2.55] | ***** |
| **6E** | Female 1000lux ML vs MD | 6 | 4.084 | 0.0095 | — | **Cohen's d_z = 1.67** | [0.43, 2.90] | ****** |
| **Manuscript Fig. 6F (Diurnality Index by Sex) - within-sex condition planned; M-vs-F FDR** | | | | | | | | |
| **6F** | Male 300 vs 1000 DI | 6 | -2.888 | 0.0343 | — | **Cohen's d_z = -1.18** | [-2.22, -0.14] | ***** |
| **6F** | Female 300 vs 1000 DI | 6 | -2.978 | 0.0309 | — | **Cohen's d_z = -1.22** | [-2.27, -0.16] | ***** |
| **6F** | Male 1000 vs DD DI | 3 | 2.167 | 0.1625 | — | **Cohen's d_z = 1.25** | [-0.26, 2.76] | **ns** |
| **6F** | Female 1000 vs DD DI | 5 | 3.947 | 0.0169 | — | **Cohen's d_z = 1.77** | [0.36, 3.17] | ***** |
| **6F** | 300lux DI M vs F | 6/6 | -0.322 | 0.7539 | 0.7539 | **Hedges' g = -0.17** | [-1.22, 0.88] | **ns** |
| **6F** | 1000lux DI M vs F | 6/6 | 1.522 | 0.1591 | 0.3182 | **Hedges' g = 0.81** | [-0.28, 1.90] | **ns** |
| **Manuscript Fig. 6G (Anticipation by Sex) - within-condition Day-vs-Dusk & Night-vs-Dawn planned; M-vs-F FDR** | | | | | | | | |
| **6G** | Male 300lux Day vs Dusk | 6 | -7.855 | 0.0005 | — | **Cohen's d_z = -3.21** | [-5.19, -1.22] | ******* |
| **6G** | Male 300lux Night vs Dawn | 6 | -3.593 | 0.0157 | — | **Cohen's d_z = -1.47** | [-2.62, -0.31] | ***** |
| **6G** | Male 1000lux Day vs Dusk | 6 | -0.182 | 0.8629 | — | **Cohen's d_z = -0.07** | [-0.88, 0.73] | **ns** |
| **6G** | Male 1000lux Night vs Dawn | 6 | -3.604 | 0.0155 | — | **Cohen's d_z = -1.47** | [-2.63, -0.32] | ***** |
| **6G** | Male DD Day vs Dusk | 3 | -6.384 | 0.0237 | — | **Cohen's d_z = -3.69** | [-6.85, -0.53] | ***** |
| **6G** | Male DD Night vs Dawn | 3 | -7.352 | 0.0180 | — | **Cohen's d_z = -4.24** | [-7.82, -0.66] | ***** |
| **6G** | Female 300lux Day vs Dusk | 6 | -2.745 | 0.0405 | — | **Cohen's d_z = -1.12** | [-2.14, -0.10] | ***** |
| **6G** | Female 300lux Night vs Dawn | 6 | -1.684 | 0.1530 | — | **Cohen's d_z = -0.69** | [-1.58, 0.20] | **ns** |
| **6G** | Female 1000lux Day vs Dusk | 6 | -2.216 | 0.0775 | — | **Cohen's d_z = -0.90** | [-1.85, 0.05] | **ns** |
| **6G** | Female 1000lux Night vs Dawn | 6 | -2.149 | 0.0843 | — | **Cohen's d_z = -0.88** | [-1.82, 0.06] | **ns** |
| **6G** | Female DD Day vs Dusk | 5 | -4.765 | 0.0089 | — | **Cohen's d_z = -2.13** | [-3.72, -0.55] | ****** |
| **6G** | Female DD Night vs Dawn | 5 | 1.016 | 0.3670 | — | **Cohen's d_z = 0.45** | [-0.47, 1.38] | **ns** |
| **6G** | 1000lux Dusk M vs F | 6/6 | 0.656 | 0.5268 | 0.5268 | **Hedges' g = 0.35** | [-0.70, 1.40] | **ns** |
| **6G** | 1000lux Dawn M vs F | 6/6 | 0.919 | 0.3798 | 0.5268 | **Hedges' g = 0.49** | [-0.57, 1.55] | **ns** |
| **Manuscript Fig. S4 (Free-Running Period by Sex) - secondary** | | | | | | | | |
| **S4** | Tau Male vs Female | 3/5 | 1.061 | 0.3296 | — | **Hedges' g = 0.67** | [-0.61, 1.96] | **ns** |

**Supplementary Table 1. Effect sizes and 95% confidence intervals for primary statistical comparisons.** For each primary comparison, the test statistic, uncorrected p-value, Benjamini-Hochberg false-discovery-rate (FDR) adjusted p-value (q-value) where applicable, standardized effect size, and 95% confidence interval are reported. Paired comparisons use Cohen's d_z (mean of within-animal differences divided by their standard deviation); independent comparisons use Hedges' g (bias-corrected for small sample size); the one-sample free-running-period comparison uses Cohen's d. Confidence intervals are normal-approximation. Multiple-comparisons correction was applied selectively according to the role of each analysis (see note below): pre-specified primary hypotheses are reported as planned comparisons without correction (q column shown as —), whereas post-hoc secondary and exploratory comparisons are FDR-corrected within their family. The significance column (Sig.) reflects the p-value appropriate to each analysis: uncorrected for planned comparisons, FDR-adjusted for post-hoc/exploratory comparisons. *** < 0.001, ** < 0.01, * < 0.05, ns = not significant. All values were computed directly from the per-animal data in MATLAB.

Supplementary Table 2:

| **Animal** | **Condition** | **MESOR** | **24-h Amp.** | **Acrophase (h)** | **12-h Amp.** | **Crepusc. ratio** | **IS** |
| --- | --- | --- | --- | --- | --- | --- | --- |
| **AO1** | 300 lux | 264,512 | 71,431 | 10.54 | 90,109 | 1.261 | 0.340 |
| **AO2** | 300 lux | 218,809 | 70,678 | 10.52 | 72,892 | 1.031 | 0.383 |
| **AO3** | 300 lux | 231,724 | 49,912 | 11.48 | 57,275 | 1.148 | 0.353 |
| **AO4** | 300 lux | 239,904 | 53,791 | 6.10 | 54,603 | 1.015 | 0.213 |
| **AO5** | 300 lux | 324,988 | 129,748 | 8.00 | 48,555 | 0.374 | 0.318 |
| **AO6** | 300 lux | 256,013 | 109,310 | 10.04 | 62,027 | 0.567 | 0.280 |
| **AO7** | 300 lux | 416,813 | 222,264 | 8.83 | 99,850 | 0.449 | 0.339 |
| **AO8** | 300 lux | 368,546 | 115,839 | 8.95 | 185,063 | 1.598 | 0.436 |
| **AO9** | 300 lux | 248,611 | 31,311 | 7.83 | 121,184 | 3.870 | 0.321 |
| **AO10** | 300 lux | 143,425 | 42,113 | 7.01 | 75,297 | 1.788 | 0.220 |
| **AO11** | 300 lux | 180,096 | 112,622 | 11.67 | 107,517 | 0.955 | 0.714 |
| **AO12** | 300 lux | 246,013 | 186,551 | 9.38 | 181,298 | 0.972 | 0.599 |
| **AO1** | 1000 lux | 428,349 | 263,676 | 5.96 | 63,263 | 0.240 | 0.364 |
| **AO2** | 1000 lux | 185,234 | 67,380 | 8.27 | 64,448 | 0.956 | 0.314 |
| **AO3** | 1000 lux | 560,240 | 468,678 | 5.82 | 77,413 | 0.165 | 0.805 |
| **AO4** | 1000 lux | 205,602 | 60,108 | 5.85 | 7,749 | 0.129 | 0.272 |
| **AO5** | 1000 lux | 429,852 | 276,660 | 5.60 | 70,541 | 0.255 | 0.586 |
| **AO6** | 1000 lux | 279,623 | 119,211 | 9.01 | 47,982 | 0.403 | 0.421 |
| **AO7** | 1000 lux | 678,228 | 469,387 | 6.42 | 94,110 | 0.200 | 0.681 |
| **AO8** | 1000 lux | 411,941 | 185,152 | 6.33 | 229,957 | 1.242 | 0.496 |
| **AO9** | 1000 lux | 683,019 | 550,559 | 5.78 | 112,691 | 0.205 | 0.860 |
| **AO10** | 1000 lux | 395,337 | 216,673 | 7.11 | 102,621 | 0.474 | 0.515 |
| **AO11** | 1000 lux | 152,449 | 127,126 | 8.94 | 84,518 | 0.665 | 0.431 |
| **AO12** | 1000 lux | 103,644 | 45,953 | 6.64 | 28,938 | 0.630 | 0.197 |
| **AO5** | DD | 185,666 | 71,971 | 12.05 | 121,023 | 1.682 | 0.313 |
| **AO6** | DD | 215,069 | 115,232 | 11.01 | 124,706 | 1.082 | 0.418 |
| **AO7** | DD | 374,926 | 121,267 | 8.70 | 78,172 | 0.645 | 0.173 |
| **AO8** | DD | 358,089 | 166,644 | 9.97 | 142,905 | 0.858 | 0.368 |
| **AO9** | DD | 299,496 | 50,325 | 10.18 | 50,207 | 0.998 | 0.129 |
| **AO10** | DD | 561,983 | 163,626 | 12.41 | 143,327 | 0.876 | 0.398 |
| **AO11** | DD | 416,518 | 344,863 | 10.81 | 218,290 | 0.633 | 0.699 |
| **AO12** | DD | 365,330 | 126,099 | 7.70 | 103,630 | 0.822 | 0.428 |

**Supplementary Table 2. Per-animal Cosinor parameters and interdaily stability by lighting condition.**

For each animal and lighting condition, the pooled mean hourly activity profile (selected pixel difference; last 14 days for 300 and 1000 lux, last 7 days for constant darkness) was fit with a bimodal Cosinor model (24-hr + 12-hr harmonic). MESOR is the rhythm-adjusted mean; 24-h amplitude and acrophase describe the dominant diurnal component (acrophase in ZT hours for 300/1000 lux and CT hours for constant darkness, DD); 12-hr amplitude describes the crepuscular harmonic; the crepuscularity ratio is the 12-hr:24-hr amplitude ratio (higher values indicate more pronounced crepuscular structure). Interdaily stability (IS) is a nonparametric measure of day-to-day reproducibility of the 24-hr activity pattern (range 0-1; higher values indicate a more stable rhythm). Constant-darkness values are available for the eight animals that experienced that condition.

**Summary (mean ± SD across animals).** 300 lux: 24-hr amplitude 99,631 ± 58,937, acrophase ZT9.20 ± 1.75, crepuscularity ratio 1.25 ± 0.93, IS 0.38 ± 0.15 (n = 12). 1000 lux: 24-hr amplitude 237,547 ± 174,222, acrophase ZT6.81 ± 1.25, crepuscularity ratio 0.46 ± 0.35, IS 0.50 ± 0.21 (n = 12). Constant darkness (DD): 24-hr amplitude 145,003 ± 90,130, acrophase CT10.35 ± 1.59, crepuscularity ratio 0.95 ± 0.33, IS 0.37 ± 0.18 (n = 8).

**Within-animal comparisons (300 vs 1000 lux, paired t-tests).** Bright light significantly increased 24-hr amplitude (p = 0.028), advanced the acrophase of the dominant diurnal component by approximately 2.4 hr (p < 0.001), and reduced the crepuscularity ratio (p = 0.019). The 12-hr amplitude itself did not differ significantly (p = 0.35), and interdaily stability did not differ significantly between conditions (p = 0.17).
